# Development of A Machine Learning Algorithm to Classify Drugs Of Unknown Fetal Effect

**DOI:** 10.1038/s41598-017-12943-x

**Published:** 2017-10-09

**Authors:** Mary Regina Boland, Fernanda Polubriaginof, Nicholas P. Tatonetti

**Affiliations:** 1 Department of Biostatistics, Epidemiology and Informatics, University of Pennsylvania, Philadelphia, USA; 2 Institute for Biomedical Informatics, University of Pennsylvania, Philadelphia, USA; 3 Center of Excellence in Environmental Toxicology, University of Pennsylvania, Philadelphia, USA; 4 Department of Biomedical and Health Informatics, Children’s Hospital of Philadelphia, Philadelphia, USA; 5 Department of Biomedical Informatics, Columbia University, New York, USA; 6 Department of Medicine, Columbia University, New York, USA; 7 Department of Systems Biology, Columbia University, New York, USA; 8 Observational Health Data Sciences and Informatics, Columbia University, New York, USA

## Abstract

Many drugs commonly prescribed during pregnancy lack a fetal safety recommendation – called FDA ‘category C’ drugs. This study aims to classify these drugs into harmful and safe categories using knowledge gained from chemoinformatics (i.e., pharmacological similarity with drugs of known fetal effect) and empirical data (i.e., derived from Electronic Health Records). Our fetal loss cohort contains 14,922 affected and 33,043 unaffected pregnancies and our congenital anomalies cohort contains 5,658 affected and 31,240 unaffected infants. We trained a random forest to classify drugs of unknown pregnancy class into harmful or safe categories, focusing on two distinct outcomes: fetal loss and congenital anomalies. Our models achieved an out-of-bag accuracy of 91% for fetal loss and 87% for congenital anomalies outperforming null models. Fifty-seven ‘category C’ medications were classified as harmful for fetal loss and eleven for congenital anomalies. This includes medications with documented harmful effects, including naproxen, ibuprofen and rubella live vaccine. We also identified several novel drugs, e.g., haloperidol, that increased the risk of fetal loss. Our approach provides important information on the harmfulness of ‘category C’ drugs. This is needed, as no FDA recommendation exists for these drugs’ fetal safety.

## Introduction

In the late 1950s thalidomide, an approved sedative, was promoted as a new modern treatment for morning sickness^1^. Thousands of pregnant women began taking the drug resulting in a dramatic increase in spontaneous abortions (i.e., ‘miscarriages’), and congenital abnormalities^2^. By mid-1961 it became clear that thalidomide was the culprit behind the observed increase in malformations. This led to the drug’s removal from the market^3^ and a permanent usage ban among women who may become pregnant. Afterwards, stringent guidelines were implemented for drugs targeted at pregnant females.

Over the years the number of medications taken by pregnant women has grown. Concern over this ‘epidemic of prescribing’ among pregnant women began in the 1970 s^4^. A Danish study found that 44.2% of women received prescriptions for at least one medication during pregnancy^5^. Anti-inflammatory drugs were commonly prescribed medications in pregnancy despite studies showing increased risk of miscarriage or fetal loss^6^. However, in many cases the effects that specific pharmacologics have on fetal outcome remains unknown. The Food and Drug Administration (FDA) lists pharmacological drugs with unknown fetal outcomes as category C (‘risk not ruled out’) while those with known teratogenic properties (such as thalidomide) are listed as category X (‘contraindicated in pregnancy’). An estimated 37.8% of pregnant women on medications received at least one FDA category C drug^7^ without having clear guidance over the potential fetal risks these medications incur. Therefore, detailed study of these enigmatic drugs is greatly needed.

Many pediatric-based research networks exist, including PEDSnet^8^. Unfortunately, these large pediatric-based clinical data research networks are insufficient for investigating the effect of maternal drug exposure on the developing fetus as they lack linked maternal-fetal records. At the same time, traditional methods that utilize post-market reporting systems to identify agents responsible for fetal anomalies and/or loss are limited, especially with regards to sample size (e.g., parents must report the anomaly to a registry)^9^. Further, many studies focus on a drug’s effect on fetal development among drugs such as doxycycline (a category D drug that is known to be harmful during fetal development)^10^. Recruiting pregnant women for participation in prospective trials is challenging even for non-pharmacological interventions, which often results in small sample sizes that may be underpowered to assess fetal risk^11,12^. EHRs were used previously to study birth-related effects^13^ with machine learning algorithms showing promise^14,15^. Many birth-related elements are available within EHRs even if sometimes access is limited^16^. Therefore, an EHR system containing linked maternal and fetal information would be the ideal dataset for an algorithm that classifies FDA category C (i.e., drugs with unknown fetal effect) into harmful and safe bins.

This study aims to systematically investigate fetal outcomes, both fetal loss and congenital anomalies, following pharmacological exposure to category C drugs. This will provide both pharmacologists and physicians with a much-needed initial classification of these ‘unknown fetal effect’ drugs. Because fetal loss and congenital anomalies are two distinct outcomes, we perform two separate retrospective cohort studies.

## Results

### Clinical Cohorts

We extracted females with live-born births at Columbia University Medical Center (CUMC) - New York Presbyterian Hospital (NYPH) or CUMC-NYPH where data on maternal drug exposure was captured in the Electronic Health Record (EHR) system. This means that the female had at least one prescription recorded in the EHR within a 1.3-year period prior to the child’s birthdate. Infants with congenital anomalies were identified as those having a diagnosis within 90 days of life. The resulting dataset contained 31,240 pregnancies resulting in a live birth without a congenital anomaly and 5,658 pregnancies with a congenital anomaly (either major or minor). This cohort is referred to as the ‘congenital anomaly’ cohort while the cohort containing the subset with minor anomalies is referred to as the ‘minor congenital anomaly’ cohort. Of pregnancies with a recorded anomaly, 1,588 had a minor anomaly. Demographics of all pregnant females in both cohorts are given in Table 1. We obtained approval for this study from CUMC’s Institutional Review Board.

**Table 1:** Demographics of Pregnant Females Included in Study

Demographic	Fetal Loss Dataset			Congenital Anomaly Dataset		
	Without Fetal Loss (N = 33043)	With Fetal Loss (N = 14922)	P	Without Congenital Anomaly (N = 31240)	With Congenital Anomaly (N = 5658)	P
**Ethnicity**						
Hispanic	13060 (39.5%)	6558 (43.9%)	0.215	12721 (40.7%)	2055 (36.3%)	0.123
Not-Reported as Hispanic	19983 (60.5%)	8364 (56.1%)		18519 (59.3%)	3603 (63.7%)	
**Race**			0.226			
Asian	877 (2.65%)	147 (0.99%)		755 (2.42%)	148 (2.62%)	0.076
Black	3131 (9.48%)	1448 (9.70%)		2871 (9.19%)	539 (9.53%)	
Other*	9776 (29.59%)	4054 (27.17%)		9041 (31.29%)	1810 (31.99%)	
Unknown**	8580 (26.0%)	5775 (38.7%)		8672 (27.8%)	1634 (28.9%)	
White	10679 (32.3%)	3498 (23.4%)		9901 (31.7%)	1527 (27.0%)	
**Age at birth/fetal loss (median and first-third quartile)**	29.28 (24.03–34.40)	27.54 (22.76–33.46)	<0.001	28.92 (23.80–34.14)	29.27 (24.15–34.25)	0.382

^*^For privacy purposes ‘Other’ includes Indian, Pacific Islander and Other. P-values were calculated before merging.

**Unidentified/Declined/Unknown.

For the ‘fetal loss’ cohort, we selected patients with any recorded fetal loss/death as indicated by a diagnosis within the International Classification of Diseases, 9^th^ edition (ICD-9) range 630–639. For controls, patients were selected with no prior fetal loss in their records and having at least one single live birth recorded at NYPH. This resulted in a dataset of 14,922 pregnancies with fetal loss and 33,043 pregnancies without fetal loss. The most frequent fetal loss diagnoses are provided in Table S1.

### Pharmacological Drug Dataset

The FDA pregnancy categories and their descriptions along with the distinct number of drugs belonging to each are given in Table S2. The most common category was category ‘C: Risk Not Ruled Out’ followed by the lower risk category ‘B: No Risk in Other Studies’. We also extracted the ATC first-level categories for all distinct drugs included in our analysis. The most common categories were ‘Alimentary Tract and Metabolism’ followed by ‘Dermatologicals’ (Table S3). Drugs that were commonly prescribed with legal termination were identified as these could bias our fetal loss results. Supplementary Dataset 1 contains a list of all drugs where at least 2% of women were first prescribed the drug the same day as a legal termination. These are ‘drugs typically prescribed with legal termination’. Two drugs used in chemical abortions: Mifepristone (200 MG) and Misoprostol (0.2 MG)^17^ were commonly first prescribed to women at CUMC-NYPH with legal termination (15.1% and 14.6% respectively).

### Classifying FDA Category C Drugs As ‘Harmful or ‘Safe’

#### Logistic Regression

We constructed a logistic regression model with a binary outcome variable representing a not-known-to-be-harmful pregnancy classification (FDA category A or B), hereafter referred to as ‘safe’ versus a severe pregnancy classification (FDA category D or X), hereafter referred to as ‘harmful’. For both congenital anomaly models (i.e., all anomalies, and minor anomalies), we added all possible features that could inform the model. The odds ratios along with their 95% confidence intervals (CIs) are shown (Figure S1).

#### Random Forest Classification of Category C Drugs

**Figure 1:**
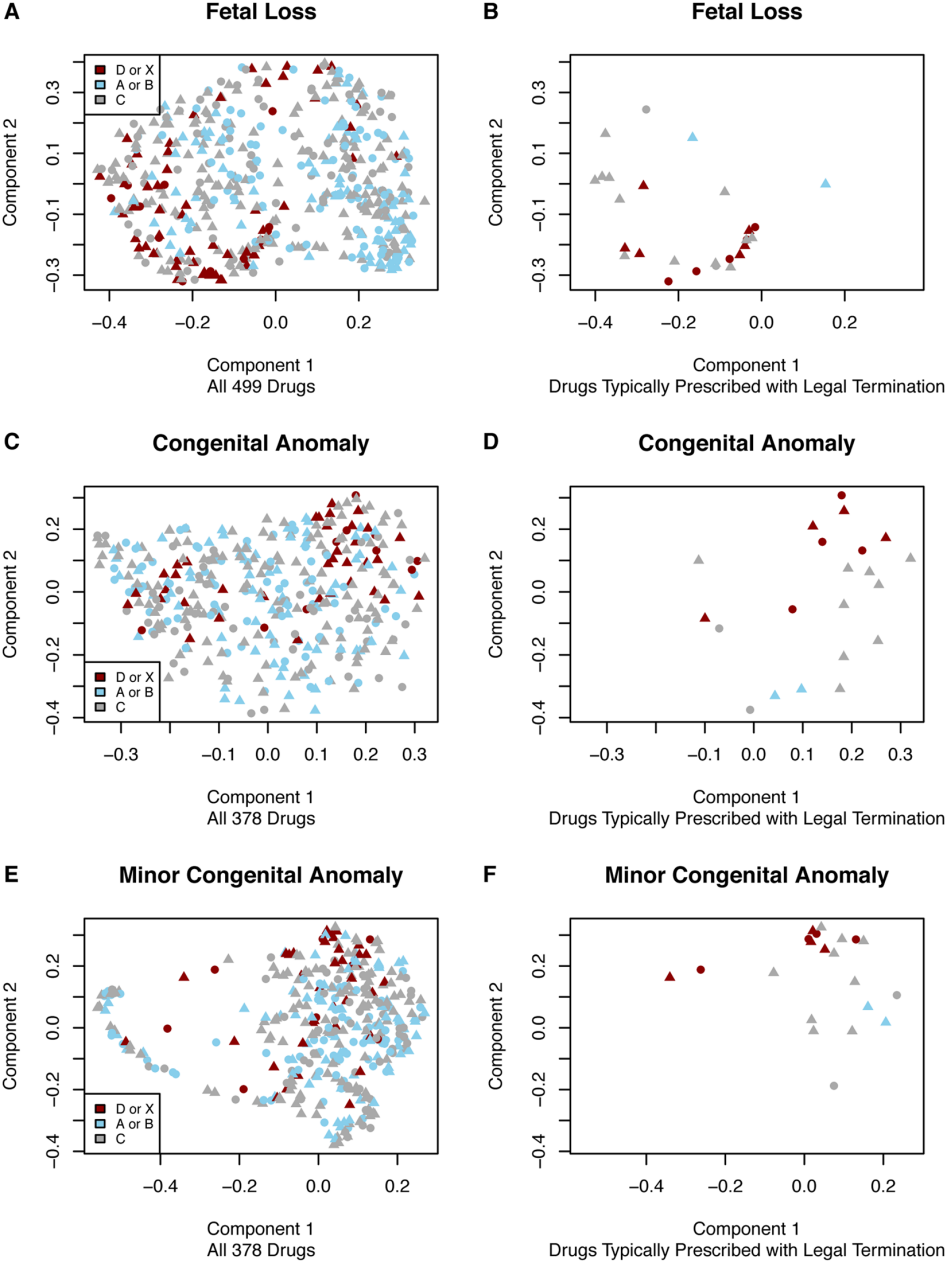
Multi-Dimensional Scaling (MDS) Component Plots for: Fetal Loss, Congenital Anomaly and Minor Congenital Anomaly. The three subplots on the left hand side of the figure (**A**,**C**,**E**) contain all drugs while on the right hand side of the figure (**B**,**D**,**F**) contain only drugs typically prescribed with legal termination. Red drugs are those shown as category D or X, blue drugs are category A or B while category C drugs are shown as grey. For all subplots, triangles indicate that the drug affected a protein encoded by a known Mendelian gene whereas circles indicate that the drug did not affect a protein encoded by a known Mendelian gene. For congenital anomalies, the proportion with an anomaly for each of the 5 exposure periods (2 pre-conception and 3 trimesters) were included as features. For fetal loss, only the 2 pre-conception periods and the first trimester were included.

A random forest model was built using the proportion with anomaly (for the congenital anomaly cohort) or the proportion with fetal loss (for the fetal loss cohort) at each trimester of exposure. The model was run with 1000 trees and we constructed a multi-dimensional scaling (MDS) component plot to illustrate the separation among drugs achieved using only the proportion with anomaly/fetal loss. Fifty-seven medications were classified as harmful and 206 safe in the fetal loss cohort. Eleven medications were classified as harmful and 181 safe in the congenital anomalies cohort. Figure 1 shows the separation between the known-harmful drugs (category D or X) in bright red from the safe drugs in light blue (category A or B). The separation between the harmful and safe drugs is most evident for the fetal loss cohort. We also separated out drugs that are used in legal termination to show where in the various plots those drugs appear. In most cases drugs prescribed during legal terminations cluster with known harmful (category D or X) drugs.

**Figure 2:**
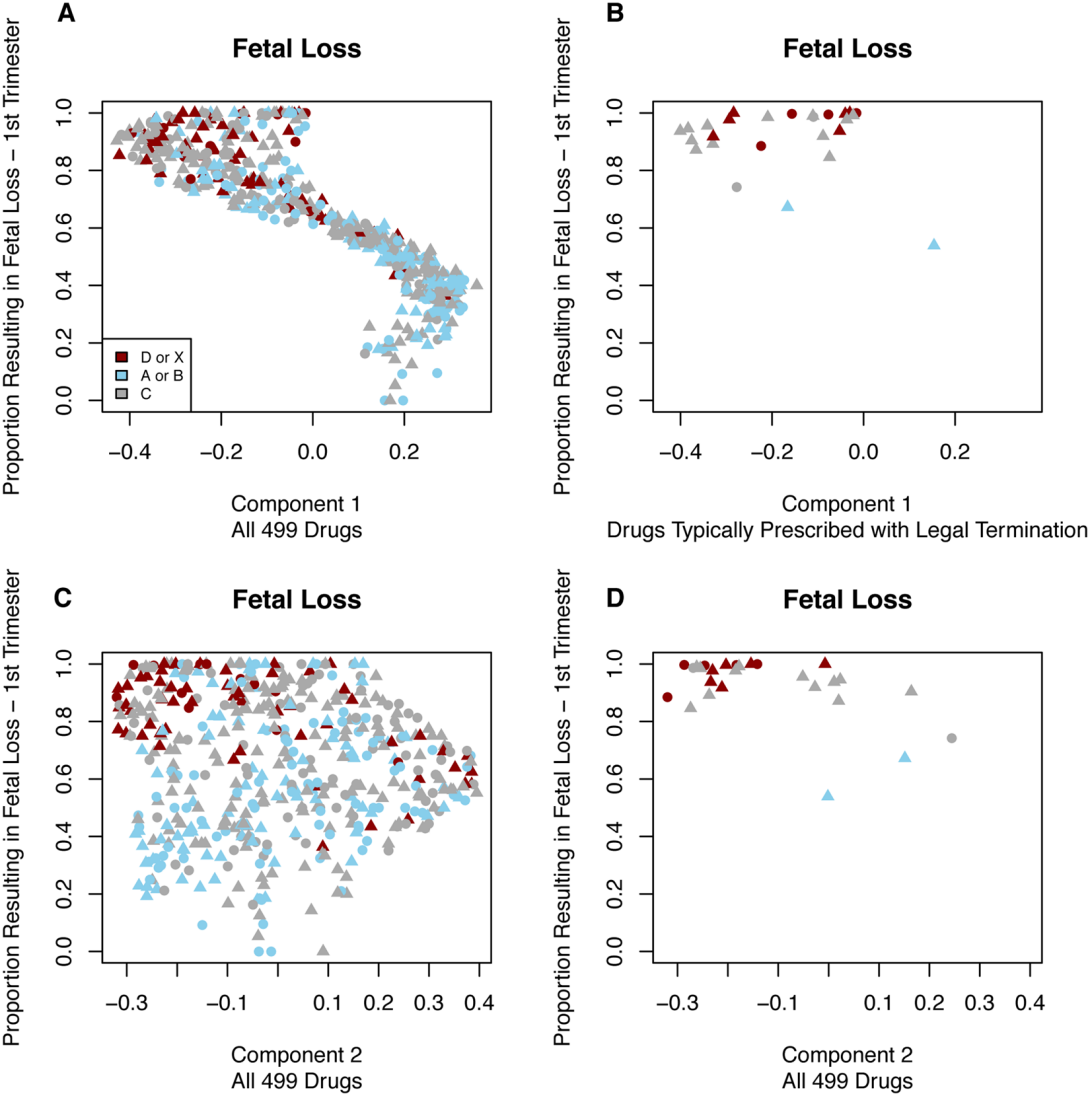
Component vs. Proportion with Fetal Loss. There is a clear relationship between the first component and the proportion of individuals experiencing fetal loss following prenatal exposure to the drug during the first trimester. This effect is not entirely due to drugs prescribed for legal termination, which are shown separately in the right most subplots. Red drugs are those shown as category D or X, blue drugs are category A or B while category C drugs are shown as grey. For all subplots, triangles indicate that the drug affected a protein encoded by a known Mendelian gene whereas circles indicate that the drug did not affect a protein encoded by a known Mendelian gene.

We observed a clear relationship between the first MDS component and the proportion of women experiencing fetal loss following first trimester drug exposure (Fig. 2). Some of this effect could be due to legal termination, so we identify those drugs empirically determined to be involved in legal termination procedures. This showed that the relationship was not solely due to drugs involved in legal termination. We also investigated the relationship between the MDS scaling components and proportions of offspring with a congenital anomaly across all three trimesters, not just the first trimester. These are shown in Figures S2–S7.

Next we ran the random forest model with all potentially informative features using 2000 trees. Each feature’s contribution to the model’s performance was assessed using Mean Decrease in Accuracy (MDA). Features with high MDA are more important in contributing to the model’s performance. We found that the number of individuals exposed to a drug at a given trimester was highly informative in predicting whether a drug was harmful or safe (Figure S8). Interestingly, the proportion born with an anomaly following maternal drug exposure during a given trimester was not as informative in the model because the known FDA class affects the exposure pattern.

Certain ATC drug classes were found to be very informative in the model (Figure S8). These include nervous system drugs (ATC: N), systemic hormonal preparations excluding sex hormones (ATC: H), anti-neoplastic and immune-modulating agents (ATC: L), genito-urinary system and sex hormones (ATC: G) and respiratory system (ATC: R) drugs. The ordering of the specific categories importance varied by model with nervous system drugs being the most informative in both the congenital anomalies (major and minor) and the minor anomalies only models.

Importantly, a binary indicator variable for whether a drug affected a vitamin-related gene (from DisGeNET) was consistently more informative then a drug being a prenatal supplement/mineral/vitamin (Figure S8). Additionally, knowing whether a drug affected or inhibited a Mendelian gene was more informative then knowing whether a drug affecting a vitamin-related gene. This indicates the importance of drugs’ Mendelian gene inhibition status.

The out-of-bag (OOB) estimated error rate was 9.36% for the fetal loss model (containing 235/499 drugs with known non-C FDA class), and 12.90% for both the congenital anomalies model (containing 186/378 drugs with known non-C FDA class) and the minor anomalies model (also containing 186/378 drugs with known non-C FDA class). The estimated accuracy was 90.64% for the fetal loss model, and 87.10% for both anomalies models. The null accuracy was 71.06% for fetal loss and 75.27% for congenital anomalies. Our models outperformed the null with p-values of 4.95 × 10^-4^ and 0.0465 respectively. Supplementary Dataset 2–4 containing the prediction results for all category C drugs along with features (a dataset per outcome).

**Figure 3:**
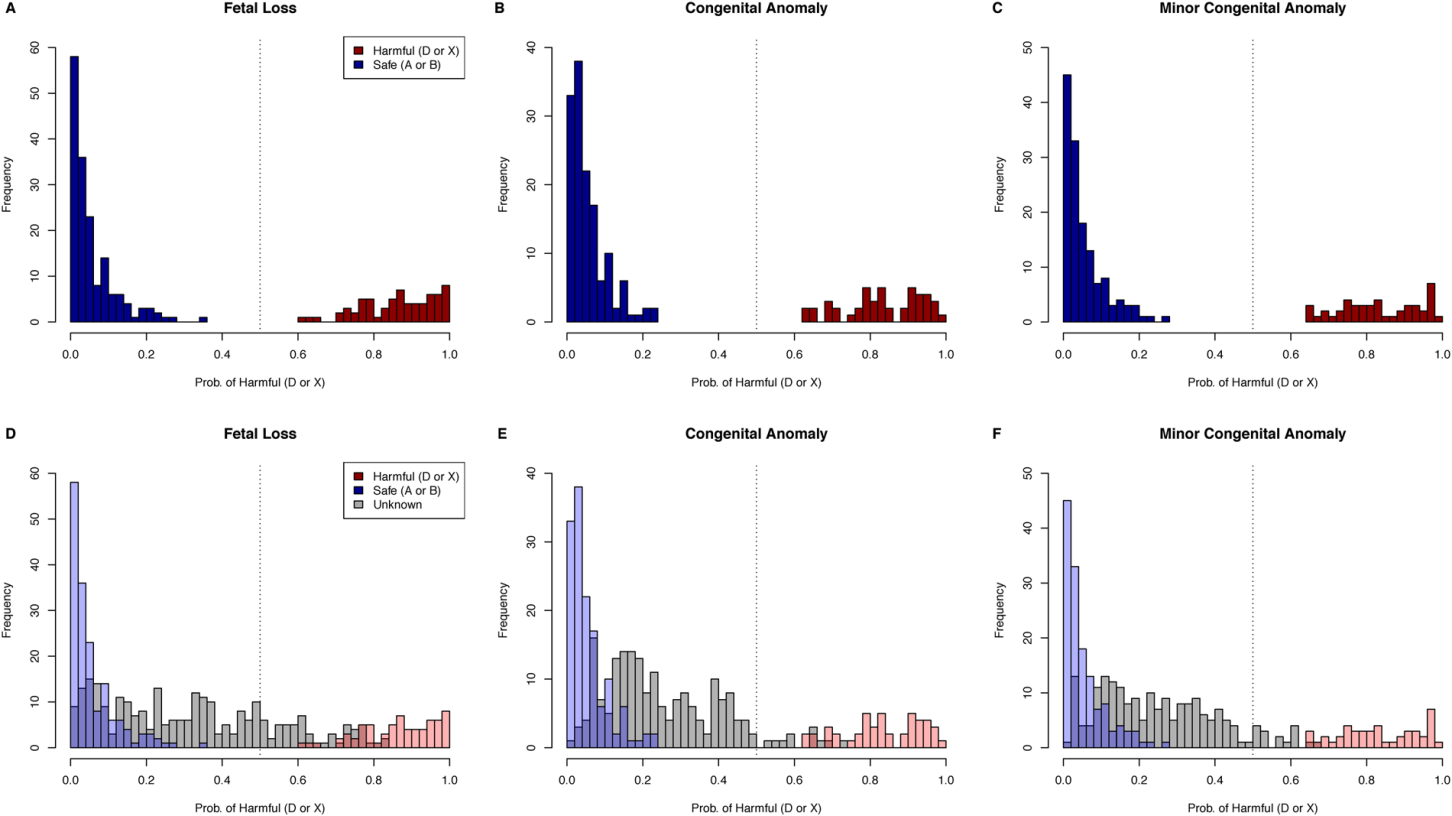
Model Probability of Being a Harmful Drug (D or X). The top portion of the graph shows drugs with known FDA pregnancy class. All drugs above the 50% probability threshold were predicted to be harmful and were harmful (three top graphs) across all models including fetal loss, congenital anomaly and minor congenital anomalies alone. In the lower three graphs FDA category C drugs are included (depicted in light grey). These drugs have no FDA recommendation regarding their safety during pregnancy. The majority of these drugs were predicted to be pregnancy safe (less than 50% probability of being harmful). While some drugs were above the 50% threshold and were more similar to known harmful drugs.

**Figure 4:**
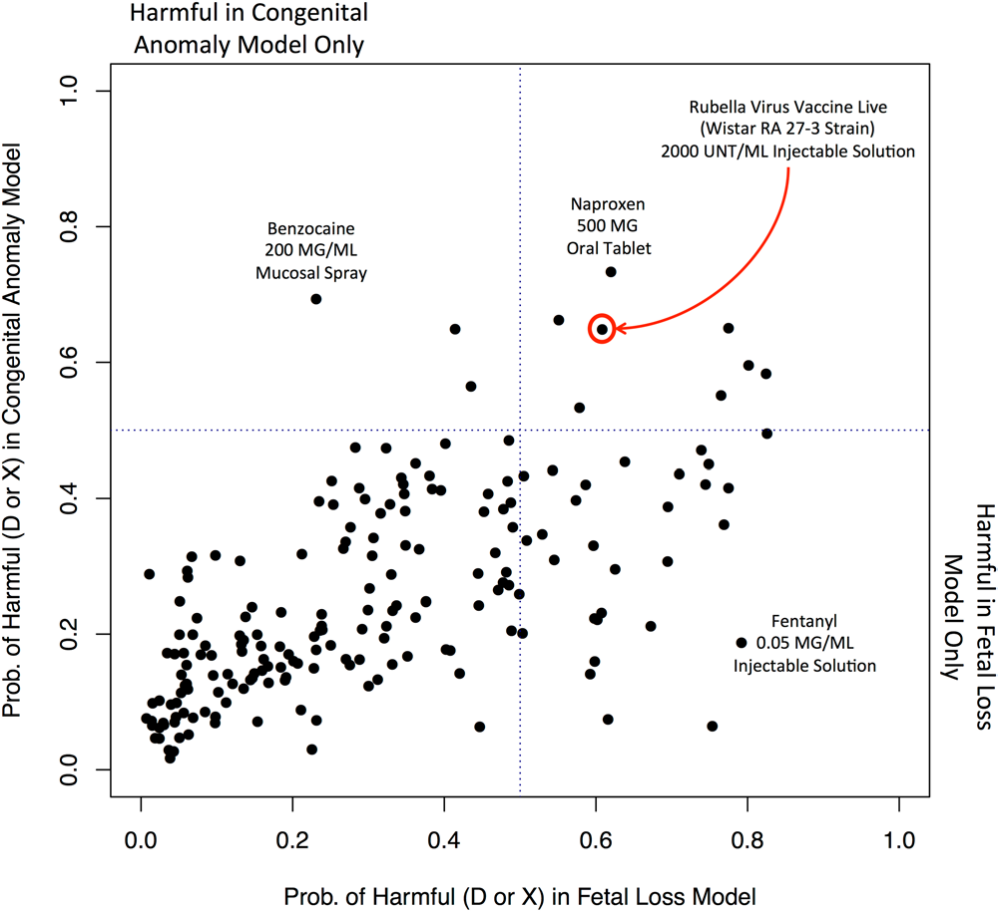
Model Probability of Being a Harmful Drug (D or X) in Congenital Anomaly Model vs. Fetal Loss Model for Category C Drugs (i.e., those with no FDA recommendation). The model probabilities for a drug’s harmful status were highly correlated (r = 0.63, p < 0.001) between both congenital anomaly and fetal loss models. NSAIDs like naproxen were predicted harmful by both models. Also live rubella vaccination was harmful in both models. Other drugs were predicted harmful in increasing the risk of either fetal loss only (lower right hand quadrant) or congenital anomalies only (upper left hand quadrant). These may require further investigation to determine the mechanistic rationale for their predicted harm in one fetal outcome over the other.

Drugs predicted to be harmful in the fetal loss model are displayed in Table 2 (Overall OOB accuracy: 90.64%) and Table 3 shows drugs predicted to be harmful in the congenital anomaly model (Overall OOB accuracy: 87.10%). All known harmful drugs (D or X) had a model probability_harmful_ above 50% while all not-known-to-be-harmful (or safe) drugs (A or B) had a model probability_harmful_ below 50% (Fig. 3). Category C drugs, where the FDA gives no pregnancy recommendation, had probabilities of being harmful across a large spectrum (Fig. 3). All 192 category C drugs included in the congenital anomalies cohort were also in the fetal loss cohort (fetal loss model contained 264 category C drugs). This allowed us to compare the probability that a drug was harmful in increasing the risk of fetal loss and congenital anomalies. These two probabilities were highly correlated (r = 0.63, p < 0.001). Drugs like rubella virus vaccine were predicted harmful in increasing the risk of fetal loss and also congenital anomalies (Fig. 4). Some drugs, like Fentanyl and Benzocaine were only predicted harmful in one model. These drugs require further investigation to determine if there is a mechanistic reason for this difference.

**Table 2:** Category C Drugs Predicted to be Harmful (D or X): Fetal Loss Cohort.

Drug Name	Percent With Fetal Loss t_-2_	Percent With Fetal Loss t_-1_	Percent With Fetal Loss t_1_
*Alimentary Tract and Metabolism (ATC: A)*			
Sodium Chloride 0.0769 MEQ/ML Injectable Solution	36.8	50	73.9
3 ML Sodium Chloride 9 MG/ML Prefilled Syringe	68.8	90.5	74.1
Calcium Chloride 0.001 MEQ/ML/Glucose 50 MG/ML/Potassium Chloride 0.004 MEQ/ML/Sodium Chloride 0.103 MEQ/ML/Sodium Lactate 0.028 MEQ/ML Injectable Solution	39.8	57	87.4
Magnesium Oxide 400 MG Oral Tablet	58.3	50	81.3
Calcium Gluconate 100 MG/ML Injectable Solution	54.5	52.2	82.5
Potassium Chloride 0.4 MEQ/ML Injectable Solution	41.7	46.7	88.1
Potassium Chloride 10 MEQ Extended Release Oral Tablet	36.4	55.6	86.4
Magnesium Hydroxide 80 MG/ML Oral Suspension	38.8	53.5	92.3
Calcium Chloride 0.0014 MEQ/ML/Potassium Chloride 0.004 MEQ/ML/Sodium Chloride 0.103 MEQ/ML/Sodium Lactate 0.028 MEQ/ML Injectable Solution	43.6	47.6	87.2
*Cardiovascular System (ATC: C)*			
Dexamethasone 4 MG Oral Tablet	50	81.8	91.3
***Hydrocortisone 25 MG/ML Topical Cream***	50	72.7	70
***Ibuprofen 800 MG Oral Tablet***	59.3	71.8	99
Nifedipine 10 MG Oral Capsule	36.7	72.7	96.2
24 HR Nifedipine 30 MG Extended Release Oral Tablet	36.7	60	86.2
24 HR Nifedipine 60 MG Extended Release Oral Tablet	50	40	89.5
Furosemide 20 MG Oral Tablet	50	33.3	85.2
Labetalol hydrochloride 5 MG/ML Injectable Solution	23.5	28.6	88.5
*Genitourinary System and Sex Hormones (ATC: G)*			
Naproxen 500 MG Delayed Release Oral Tablet	47.8	43.8	84.6
***Naproxen 500 MG Oral Tablet***	46	49.1	83.3
***Dinoprostone 10 MG Drug Implant [Cervidil]***	43.9	73.1	100
1 ML Carboprost 0.25 MG/ML Injection [Hemabate]	56.3	85.7	100
Methylergonovine Maleate 0.2 MG Oral Tablet	53.3	75	99.1
Methylergonovine Maleate 0.2 MG Oral Tablet [Methergine]	45.9	75	100
Methylergonovine Maleate 0.2 MG/ML Injectable Solution [Methergine]	45.2	57.1	97.9
*Pain-Reliever Combination Drugs (ATC: N)*			
Acetaminophen 300 MG/Hydrocodone Bitartrate 10 MG Oral Tablet	77.8	100	99.2
Acetaminophen 325 MG/Codeine Phosphate 30 MG Oral Capsule	51.9	60.9	98.6
Acetaminophen 325 MG/Oxycodone Hydrochloride 5 MG Oral Tablet	36.6	45.3	90.4
Acetaminophen 650 MG Rectal Suppository [Acephen]	60.3	61.4	85.8
*Anti-Depressant or Anti-Psychotic (ATC: N)*			
Sertraline 100 MG Oral Tablet	60	41.7	68.4
Sertraline 25 MG Oral Tablet	71.4	50	75
Sertraline 50 MG Oral Tablet	53.3	41.7	76.2
Prochlorperazine 10 MG Oral Tablet	76.9	85.7	73.7
Prochlorperazine 5 MG Oral Tablet	75	64.3	84.6
Citalopram 20 MG Oral Tablet	27.3	29.4	71.4
Haloperidol 5 MG Oral Tablet	40	70	78.3
Haloperidol 5 MG/ML Injectable Solution	22.2	50	78.6
Fluoxetine 20 MG Oral Capsule	41.7	50	91.7
Trazodone Hydrochloride 50 MG Oral Tablet	57.1	38.9	45
*Migraines or Anti-Seizure (ATC: N)*			
Sumatriptan 25 MG Oral Tablet	62.5	65	75
Gabapentin 300 MG Oral Capsule	40	44.4	57.1
*Sedative (ATC: N)*			
Zolpidem tartrate 10 MG Oral Tablet	37.5	62.5	84.1
Zolpidem tartrate 5 MG Oral Tablet	39.8	49.8	90.3
*Opioid (ATC: N)*			
***Butorphanol Tartrate 2 MG/ML Injectable Solution***	62.2	75	83.3
***Hydromorphone Hydrochloride 2 MG Oral Tablet [Dilaudid]***	45	53.8	69.6
***Hydromorphone Hydrochloride 2 MG/ML Injectable Solution***	55.4	48.6	88
1 ML Hydromorphone Hydrochloride 1 MG/ML Injection	47.1	42.5	91.6
Morphine Sulfate 10 MG/ML Injectable Solution	14.3	61.5	90
Morphine Sulfate 2 MG/ML Injectable Solution	41.7	48.5	84.9
Morphine Sulfate 4 MG/ML Injectable Solution	50.9	54.7	90.9
12 HR Oxycodone Hydrochloride 10 MG Extended Release Oral Tablet	27.3	50	100
Oxycodone Hydrochloride 5 MG Oral Tablet	57.4	49.5	85
Tramadol hydrochloride 50 MG Oral Tablet	28.6	33.3	75
Fentanyl 0.05 MG/ML Injectable Solution	70.6	62.1	98.7
*Respiratory System (ATC: R)*			
Promethazine Hydrochloride 25 MG Oral Tablet	96.2	89.4	97.7
*Musculoskeletal/Sensory System (ATC: M and S)*			
1 ML Ketorolac Tromethamine 30 MG/ML Prefilled Syringe	46.4	47.9	91
*Various Systems (ATC: V)*			
Naloxone Hydrochloride 0.4 MG/ML Injectable Solution	55.6	57.1	96.1
*Vaccine*			
***Rubella Virus Vaccine Live (Wistar RA 27–3 Strain) 2000 UNT/ML Injectable Solution***	37	47.5	96.4

***Bold italics*** indicates that drug was implicated in adverse fetal outcomes for both the fetal loss model and the congenital anomaly model.

t_-2_: Pre-conception effect: −6 to −3 months before conception.

t_-1_: Pre-conception effect: −3 to 0 months before conception.

t_1_: First Trimester.

**Table 3:** Category C Drugs Predicted to be Harmful (D or X): Congenital Anomalies Cohort.

Drug Name	Percent With Anomaly t_-2_	Percent With Anomaly t_-1_	Percent With Anomaly t_1_	Percent With Anomaly t_2_	Percent With Anomaly t_3_	Period At Risk
*Predicted Harmful and Having an Increased Risk of Congenital Anomalies*						
*Vaccine*						
***Rubella Virus Vaccine Live (Wistar RA 27–3 Strain) 2000 UNT/ML Injectable Solution***	12.6	8.5	20	0	15	1^st^ or 3^rd^
*NSAID*						
Ibuprofen 200 MG Oral Tablet	12.2	18.6	29.2	0	0	1^st^
***Ibuprofen 800 MG Oral Tablet***	16.9	14.1	20	12.5	0	1^st^
Naproxen 250*** MG*** Oral Tablet	14.3	8	50	0	0	1^st^
***Naproxen 500 MG Oral Tablet***	12.9	13.2	7.7	20	0	2^nd^
*Predicted Harmful Resulting from Modified Clinical Prescribing Patterns During Pregnancy (i.e., restricted drug exposure during certain trimesters)*						
*Opioid*						
***Butorphanol Tartrate 2 MG/ML Injectable Solution***	5	14.3	0	0	10.4	*3^rd^
***Hydromorphone Hydrochloride 2 MG Oral Tablet [Dilaudid]***	9.1	14.3	0	0	0	RGDP
***Hydromorphone Hydrochloride 2 MG/ML Injectable Solution***	10.7	9.5	0	6.1	12.8	3^rd^
*Pain Reliever*						
Benzocaine 200 MG/ML Mucosal Spray	13.4	10.7	0	0	0	RGDP
*Steroid*						
***Hydrocortisone 25 MG/ML Topical Cream***	57.1	12.5	0	5.6	3.9	RGEP, decreased risk 3^rd^
*Cervical Implant*						
***Dinoprostone 10 MG Drug Implant [Cervidil]***	9.2	16.7	0	0	13.5	RGEP, decreased risk 3^rd^

***Bold italics*** indicate that drug was implicated in adverse fetal outcomes for both the fetal loss model and the congenital anomaly model.

*Most exposures (94%) occurred during the 3^rd^ trimester

RGDP: **R**arely **G**iven **D**uring **P**regnancies ending in live-born infants. The dramatic drop off in prescribing throughout the entire pregnancy (t1-t3) is why these drugs were labeled as likely to be harmful during pregnancy (D or X) due to similar patterns being observed for drugs that are known to be harmful.

RGEP: **R**arely **G**iven **E**arly in **P**regnancies ending in live-born infants. These are medications that were not given early on in the pregnancy but were given later in the pregnancy (2^nd^-3^rd^ trimester). This indicates that these medications may be harmful early on in the pregnancy, which resulted in clinicians modifying their behavior to reduce risk to the fetus during the early-developmental stages. However, in both cases above (dinoprostone and hydrocortisone) showed decreased risk of anomalies following 3^rd^ trimester exposure.

t_-2_: Pre-conception effect: −6 to −3 months before conception.

t_-1_: Pre-conception effect: −3 to 0 months before conception.

t_1_: First Trimester.

t_2_: Second Trimester.

t_3_: Third Trimester.

**Figure 5:**
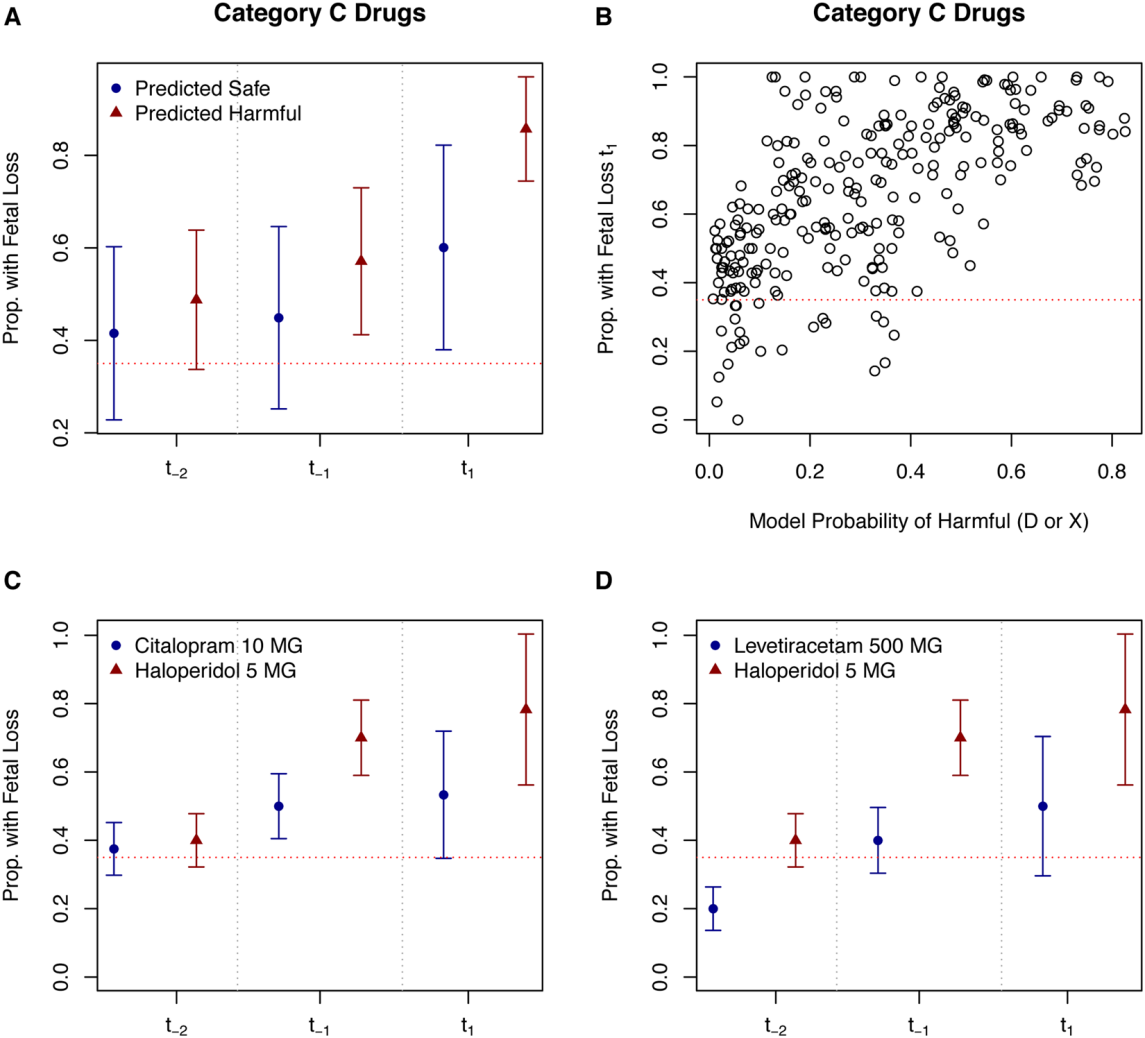
Model Probability of Being a Harmful Drug (D or X) in the Fetal Loss Model vs. Proportion with Fetal Loss: Investigation of Nervous System Medications that Are Predicted Harmful vs. Predicted Safe and Affect On Risk of Fetal Loss. The overall average proportion of fetal loss for all predicted safe and harmful medications across the different trimester exposure points are presented (upper left-hand plot). Predicted harmful drugs (probability of harmful >=0.50) increased the risk of fetal loss especially following first trimester exposure (upper right-hand plot). The anti-depressant Haloperidol 5 MG, predicted harmful by our model, is compared to the SSRI anti-depressant medication – Citalopram 10 MG, predicted safe by our model, with the former experiencing greater rates of fetal loss following exposure (lower left-hand plot). Haloperidol 5 MG is also compared to the epilepsy medication Levetiracetam 500 MG, predicted safe by our model, with haloperidol again experiencing greater rates of fetal loss following exposure (lower right-hand plot). For drugs predicted as harmful in the fetal loss model, there was an increase in fetal loss rates following exposures when compared to those predicted as safe. Red dashed horizontal lines indicate the CDC reported background rate of 35% fetal loss.

For the fetal loss model, there was a clear and intuitive relationship between the proportion with fetal loss during the first trimester and the model’s prediction that the drug was harmful (Fig. 5). Nervous system drugs in general (ATC: N) were more likely to be classified as being harmful drugs. However, a couple of nervous system medications were classified as safe (similar to category A or B drugs) by the model. Two of these drugs: Citalopram 10 MG and Levetiracetam 500 MG are shown versus a predicted harmful drug: Haloperidol 5 MG in Fig. 5. Citalopram, like haloperidol, is an anti-depressant; however, citalopram is an Selective Serotonin Reuptake Inhibitor (SSRI) whereas haloperidol is not. Levetiracetam is used in treating epilepsy and is an anti-seizure medication. Importantly, not all nervous system category C medications were predicted as harmful to the fetus by our model.

**Figure 6:**
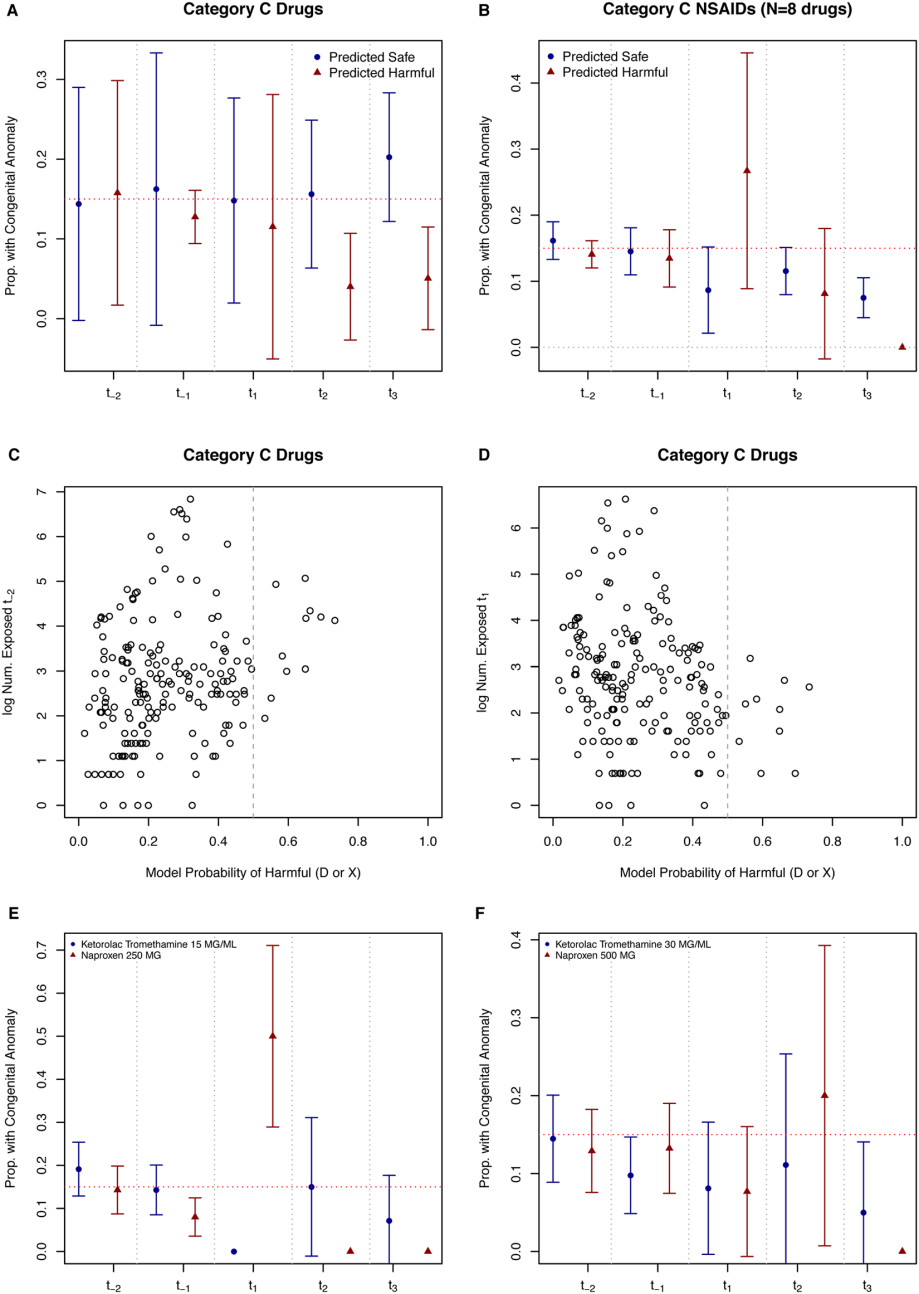
Model Probability of Being a Harmful Drug (D or X) in Congenital Anomaly Model vs. Proportion with Congenital Anomalies: Investigation of NSAIDs that Are Predicted Harmful vs. Predicted Safe and Affect on Anomaly Risk. The overall average proportion of infants with anomalies for all predicted safe and harmful medications across the different trimester exposure points are presented (upper left-hand plot). For NSAIDs, predicted harmful drugs (probability of harmful >=0.50) increased the risk of fetal loss especially following first trimester exposure (upper right-hand plot). Exposure rates changed during pregnancy for all predicted harmful category C medications. The log of the exposure rates prior to conception are shown (middle left-hand plot) and during first trimester (middle right-hand plot). There is a shift in usage patterns for those predicted as harmful. The NSAIDs naproxen 250 MG and 500 MG were both predicted harmful by our model. The NSAIDs Ketorolac Tromethanine 15 MG/ML and 30 MG/ML were both predicted as safe by our model. These two medications are compared to each other with their respective effects on risk of anomaly across the various trimesters (lower left-hand and right-hand plots). First trimester exposure to naproxen 250 MG greatly increased the risk of anomaly vs. ketorolac 15 MG/ML (lower left-hand plot). Red dashed horizontal lines indicate the CDC reported background rate of 15% for all congenital anomalies.

On the other hand, congenital anomalies are often better described in the literature, which affects treatment patterns. This was evidenced in our models as well. Overall, we did not find a clear increase of anomaly risk among our predicted harmful vs. safe medications in the anomaly models (Fig. 6). Except for our Non-Steroidal Anti-Inflammatory Drugs (NSAIDs) and rubella live vaccine findings, which showed a clear increase in anomaly risk following first trimester exposure. Importantly, our model classifies category C medications as being harmful if their features (including exposure rates and anomaly rates) are similar to known harmful medications (D or X). Therefore, several medications were prescribed during the pre-conception period but not during the first and second trimesters. Our algorithm detected these medications as being harmful while other medications were predicted to be harmful due to the increased risk of anomalies observed in our dataset. We distinguish these two types of findings in Table 3. One NSAID – Ketorolac Tromethamine – was not predicted as harmful by our model for two dosage levels. We compare this to two dosages of Naproxen, both predicted as harmful by our model, to illustrate the increased first trimester risk of anomalies for Naproxen 250 MG versus another NSAID (Fig. 6). Importantly, not all NSAIDs were predicted as harmful by our model, but only those that increased the risk of anomalies.

## Discussion

Our models successfully identified category C drugs that are likely to be harmful and those likely to be safe for fetal loss or congenital anomalies. This information is important as no prior recommendation for a drug’s effect during pregnancy was provided. This is especially true for two similar medications (e.g., two NSAIDs) with one predicted as safe (e.g., ketorolac) and the other as harmful (e.g., naproxen).

### Drugs Predicted Harmful in Congenital Anomaly Model

We predicted 11 distinct medications (eight distinct drugs) to be harmful in the congenital anomalies model. We employed a machine-learning algorithm to predict drugs that were harmful based on anomaly rates and usage patterns for drugs with known FDA pregnancy classifications. This machine learning approach predicts a drug to be harmful if one of the following conditions is met: a.) drug exposure results in a high proportion of anomalies; b.) drug usage was greatly restricted during pregnancy (i.e., females were exposed during pre-conception period at much higher rates then during pregnancy; or c.) drug was similar to known harmful drugs in terms of mechanism (e.g., ATC classification, targets proteins involved in Mendelian diseases, targets known vitamin-related genes/proteins). We clearly distinguish drugs by type in Table 3 for clarity of interpretation.

#### Non-Steroidal Anti-Inflammatory Drugs (NSAIDs)

Two predicted harmful drugs (four distinct medications) were NSAIDs, namely ibuprofen and naproxen. Several studies report an increased risk of anomalies, specifically cardiac anomalies among infants exposed to naproxen, ibuprofen, or combinations of NSAIDs^18,19^. In most cases studying the fetal effects of NSAIDs, the drugs - naproxen and/or ibuprofen - were often associated with the most number of congenital anomalies^18,20^. For both drugs, we observed the highest risk among first and second trimester exposures, with higher risk among naproxen users than ibuprofen users (Table 3). Furthermore, restriction to NSAIDs was greatly restricted during the third trimester (Fig. 6), which is consistent with current recommendations^21^. Ketorolac was classified as safe by our model and had a lower rate of anomalies especially following first trimester when compared to other NSAIDs (Fig. 6). Ketorolac is a COX-2 specific inhibitor and has been used safely in neonates and infants^22–24^.

#### Live Rubella Vaccine

Maternal exposure to live rubella vaccine was classified as harmful in both our congenital anomaly and fetal loss models (Table 2) with increased risk of fetal loss and an increased risk of anomalies. This is consistent with the literature on the harms of rubella exposure during early pregnancy^25–27^. Please note that the rubella vaccine was the only vaccine predicted to be harmful during pregnancy by our model. Prior studies demonstrate that increases in both anomalies and fetal loss were observed in women infected with rubella during pregnancy^28,29^, which we confirm in this study. However some conflicting evidence does exist regarding the fetal harm of rubella exposure^30^. We observed 96.4% of those receiving rubella vaccination in the first trimester (83 exposed during first-trimester in fetal loss cohort) resulted in a fetal loss (Table 2). This indicates the severity of first-trimester rubella exposure on fetal outcomes underscoring the importance of avoiding rubella vaccination prior to conception. It should be noted that live rubella vaccine is not indicated in pregnancy and often occurred in the pre-conception and first-trimester period of the pregnancy, indicating that the prescribing clinician was likely not aware that a pregnancy had taken place.

#### Prescribing Pattern Drop-offs During Pregnancy – Predicted Harmful in Congenital Anomaly Model

Two drugs were rarely prescribed during the entire pregnancy – Benzocaine mucosal spray and Hydromorphone Hydrochloride (Dilaudid), but were prescribed during the pre-conception period. This sudden drop-off in prescribing caused our algorithm to detect these drugs as harmful given that a similar drop-off in prescribing was observed in known harmful drugs. Hydromorphone Hydrochloride is an opioid and therefore was likely not prescribed during pregnancy given the harm that opioids have on developing fetuses^31^. The other medication – benzocaine mucosal spray – has been linked to development of methemoglobinemia in infants and because safer category B medications are available many physicians consider it contra-indicated during pregnancy^32,33^. Our machine learning approach did not know this information *a priori*, but it was able to learn this from clinician usage patterns (i.e., dramatic drop-off of prescribing during pregnancy). Several other medications were rarely used early on in the pregnancy (first and second trimester), including several opioids, and those also increased the risk of fetal loss in our fetal loss model. This was likely the reason for their contra-indication earlier on during pregnancy.

### Drugs Predicted Harmful in Fetal Loss Model

#### Drugs That May Inadvertently Induce Fetal Loss: DHCR7 Mechanism

First trimester haloperidol exposure increased the risk of fetal loss. Haloperidol injection increased risk of fetal loss from 22.2% in the 3–6 months prior to conception to 78.6% following first trimester exposure. Nine pregnancies were exposed in the 3–6 month pre-conception period while 28 pregnancies were exposed during the first trimester – 22 resulted in fetal loss. Haloperidol increases the expression of 7-dehydrocholesterol reductase (DHCR7) - an enzyme important in the conversion of 7-dehydrocholesterol to cholesterol^34^. While exposure to pharmacological DHCR7 inhibitors increases the risk of fetal anomalies, the effects of drugs that merely increase the gene’s expression are less-well known^34^. Drugs increasing DHCR7 expression are not known to increase fetal loss; however increasing DHCR7 volume would lower the amount of available 7-dehydrocholesterol used to produce vitamin D^34^. Therefore, drugs increasing DHCR7 expression could inadvertently lower maternal vitamin D levels. Patients on haloperidol have been shown to have elevated levels of 7-dehydrocholesterol^35^, which is curious as increasing DHCR7 expression would be expected to lower 7-dehydrocholesterol and elevate cholesterol (by increasing the conversion rate). Therefore, the harmful effects we observed for haloperidol could be due to the elevated 7-dehydrocholesterol levels and not a reduction in vitamin D. Further mechanism-based studies are required. In this study, we compared haloperidol to two other nervous system medications (ATC category: N), one an SSRI citalopram and the other an epilepsy medication levetiracetam. Haloperidol 5 MG tablet greatly increased the risk of fetal loss when compared to these two other nervous system FDA category C medications, which were both predicted as ‘safe’ by our model (Fig. 5C and 5D). This is important, as it might be possible for pregnant women to switch their anti-depressant medication following pregnancy.

#### Drugs Treating Symptoms of Fetal Loss

Some drugs predicted as harmful in the fetal loss cohort could have been prescribed to treat conditions leading up to a spontaneous abortion. For example, excessive bleeding often occurs during a spontaneous abortion, but a miscarriage can take several days. A drug used to treat severe bleeding following childbirth, or miscarriage, is Methylergonovine Maleate. All forms of Methylergonovine Maleate (3 different types listed in Table 2) had high rates of miscarriage following first trimester exposure – ranging from 97.9–100% of those exposed during that trimester. Typically, Methylergonovine Maleate would not be prescribed during the first trimester, unless something was wrong (e.g., excessive bleeding, which is indicative of a miscarriage). Therefore, this is likely a treatment-of-the-fetal-loss type of result. Other drugs related to fluids, including potassium chloride and calcium gluconate (ATC: A category drugs in Table 2) are likely used during fetal loss as women experience nausea while experiencing a miscarriage and would require fluids.

### Genetic Targets of Drugs More Predictive Than Classification

Prenatal vitamin supplementation is important in reducing the overall disease risk of adverse fetal effects with supplementation linked to lower rates of leukemia, pediatric brain tumors and neuroblastoma^36^. We restricted our analyses to identification of congenital anomalies diagnosed within the first 90 days of life. Therefore, we did not investigate complex outcomes such as childhood cancers or autism. However, vitamin-exposure during the prenatal period is widely considered to be important in predicting fetal outcome. All models showed that knowing whether or not a drug affected a vitamin-related protein was more important then just knowing that a drug was a prenatal supplement (Figure S8). This is important because it shows that a drug’s mechanism of action and how it interfaces with vitamin-related mechanisms is extremely important in determining fetal outcome. This was known for specific drugs^34^, but not across a larger cohort of fetal drug exposures. This knowledge can inform future fetal toxicity studies.

### Rationale for Using Logistic Regression and Random Forest

In this paper, we employed two statistical approaches: logistic regression and random forest. The logistic regression model was used only on drugs with known fetal effect (either harmful: D or X or safe: A or B). This model allowed Odds Ratios to be computed for various features included in the model among known drugs (Figure S1). This information was already known. For example, in the fetal loss model, drugs that were respiratory system drugs (ATC: R) were likely to be safe drugs whereas drugs in the systemic hormonal preparations class (ATC: H) were likely to be category D or X. We were really interested in understanding the drugs with unknown fetal effect (i.e., the category C drugs). For the purpose of classifying these unknown drugs, we developed a random forest classifier on the known drugs and then applied it to the unknown drugs to assign a probability that a drug was harmful or safe based on the information learned from the other drugs. This random forest classifier also allowed us to easily rank the importance of the features included in the model (Figure S8).

### Limitations

Our method identifies drugs predicted to be harmful given their prescribing patterns (e.g., low exposure during pregnancy), anomaly rates (e.g., proportion of exposed with an anomaly) and other chemoinformatics factors important in determining fetal outcome (e.g., affecting proteins involved in vitamin-related processes). Further study is needed to confirm drug predictions, especially for drugs that are predicted as safe to ensure that they are not harmful to the developing fetus. Some drugs may be predicted as harmful because they are prescribed during high-risk pregnancies, which are at increased risk of complications during delivery. High-risk pregnancies are known to be at a higher-risk of congenital anomalies^37^. An example of this type of finding may be Dinoprostone (or Cervidil) predicted as harmful in our congenital anomaly model. Dinoprostone is a cervical implant used to induce labor. These are often used during high-risk pregnancies^38^.

Another limitation is our exclusive use of medications recorded in EHRs. Others have investigated non-hormonal category X drugs and their prescribing patterns among pregnant women in a decision support context^39^. They found that the medication information was not of sufficient quality to construct an EHR-based alert for pregnant women^39^. We were unable to conduct a detailed chart-review for all 36,000 pregnancies to determine the accuracy of medications across the various FDA categories and drug types. Our validation of several findings on predicted harmful drugs with the literature on their effects helps to confirm our findings. However, we recognize this as a limitation of our work.

## Conclusion

In conclusion, we developed a machine learning approach that predicts drugs to be either harmful or safe in two outcome models – fetal loss and congenital anomalies. We achieved an OOB estimated accuracy of 90.6% for fetal loss and 87.1% for congenital anomalies. Some drugs were predicted as harmful because physicians stopped prescribing them upon pregnancy diagnosis – this dramatic drop-off in exposure rates triggered the algorithm to detect the drug as harmful (since a similar pattern is observed among drugs that are known to be harmful). Other drugs were predicted as harmful because of the increase in anomalies observed following exposure. Many medications predicted to be harmful by our algorithm have documented harmful effects, including naproxen, ibuprofen and rubella live vaccine. Additionally, we found that first trimester exposure to haloperidol – a drug that interferes with the DHCR7 – cholesterol – vitamin D pathway increased the risk of fetal loss. We also compare haloperidol to other nervous system medications that do not increase the risk of fetal loss to the same extent. Our approach provides much needed information for pharmacologists and prescribers interested in understanding drugs’ fetal effects and prescribing patterns in EHRs.

## Materials and Methods

### Clinical Cohorts

#### Maternal Prescription Exposure and Fetal Outcome: Live Birth

We obtained records on all infants born at the Columbia University Medical Center (CUMC) - New York Presbyterian Hospital (NYPH) healthcare system who had mothers listed in the Electronic Health Record (EHR) system. These links were created in the EHR system upon delivery to facilitate maternal-fetal care post-delivery. The EHR system contains billing information collected during routine clinical care. This information includes prescription information, diagnoses, laboratory tests and results, procedures, radiological reports and clinical free text notes. In this study, we have used only the diagnosis codes and prescription information contained with the system along with the mother-infant links. We retained all mother-infant pairs with at least one medication prescribed before birth and up to 15 months prior. Pregnant women with no medication information (e.g., not even a vitamin supplement) in the EHR system are most likely missing their medication records. Therefore, these women were not included in our analysis and only women with at least one prescribed medication, which includes vitamins, were included. We excluded all multiple infant pregnancies (e.g., twins, triplets) as these pregnancies are considered high-risk. We also excluded all pregnancies with any chromosomal abnormality diagnosed within the first three months of life (0–90 days of life). Presence of chromosomal abnormality was determined using the International Classification of Diseases, 9^th^ edition (ICD-9) range 758–758.9.

We identified infants with congenital anomalies as those having a congenital anomaly ICD-9 diagnosis, i.e., 740–759 (with 758–758.9 excluded) occurring within the first 90 days of life. Only one anomaly diagnosis was necessary for identification although some infants had multiple anomalies. We identified minor anomalies using criteria established by the New York State Department of Health, only ICD-9 codes within the 740–759 range were used^40^. For comparison purposes, the reported background rate of major congenital anomalies is 3% while the rate of minor congenital anomalies is 15% of live-born infants^41^.

#### Maternal Prescription Exposure and Fetal Outcome: Fetal Loss

All pregnancies ending in fetal loss were identified at CUMC-NYPH. Fetal loss in this study includes spontaneous abortion (i.e. ‘miscarriages’), legal/elective termination and any other forms of fetal loss/death recorded within the ICD-9 range 630–639. Because we are interested in fetal outcomes following pharmacological exposure, we only included females with at least one medication prescribed up to 15 months before fetal loss. A female may have more than one fetal loss code occurring on two separate dates (often during the course of a single hospital visit); therefore we collapsed dates to the month level. For our control population, we used women with a successful fetal outcome (e.g., single live birth) recorded at CUMC-NYPH with at least one medication prescribed up to 15 months prior to birth and who had no diagnosis of fetal loss recorded at CUMC-NYPH and whose infant was without chromosomal abnormality. According to the CDC, 17.0% of conceptions resulted in miscarriage and 18.4% ended in legal termination in 2008^42^. Because we define fetal loss to include both spontaneous abortion and legal termination, we expect a background rate of 35.4%.

### Pharmacological Drug Information

The FDA pregnancy categories for all drugs included in our study were extracted from uptodate.com^43^ and drugs.com^44^. While the FDA has recently updated this labeling system and moved away from the A-X categorization schema^45^, we chose to use it in our study because it allows researchers and physicians to easily identify drugs with unknown fetal effects (the category C drugs). If a particular drug-combo was not listed with its own FDA pregnancy category designation then we used the most severe pregnancy category from each drug in the combo. We also mapped each drug to its first-level class within the Anatomical Therapeutic Chemical (ATC) classification system, which categorizes drugs based on their organ system effects. We also extracted the Mendelian genes either inhibited or affected (regardless of mechanism) for each drug using the Online Mendelian Inheritance in Man (OMIM) (URL: https://www.omim.org/). Because drugs targeting genes involved in vitamin processes may affect fetal risk (either protective or injurious), we also identified drugs that target at least one vitamin-related gene as noted on DisGeNET – a disease-gene association network (URL: http://www.disgenet.org/).

We are interested in finding drugs that increase or decrease the risk of fetal loss following prenatal exposure. However, some medications are used to induce legal termination or to treat subsequent conditions (e.g., hemorrhage, excessive bleeding, pain). These drugs could bias our analyses; therefore, we identified drugs given to women where the first prescription of the drug was the same day as the legal termination. We calculated the proportion of legal terminations where a given prescription drug was first prescribed out of those terminations where prescription information was available. All drugs with at least 2% frequency were labeled as ‘drugs typically prescribed with legal termination’.

### Statistical Analysis

#### Identifying Trimester of Drug Exposure

For pregnancies that resulted in a single live birth, we used the average gestation period (i.e., 38 weeks) as reported by the Centers for Disease Prevention and Control (CDC)^46^. We then divided the 38-week pregnancy into three equal-sized periods (12.67 weeks each) as ‘trimesters’. For pregnancies that resulted in fetal loss, we used the average time to fetal loss. CDC reported that 91.6% of legal terminations occur within 13 weeks gestation with many other forms of fetal loss occurring prior to 13 weeks as well^47^. Therefore, an exposure could have only occurred during the first trimester (i.e., one 12.67 week period). We also investigated two pre-conception periods (each 3 months in size) where exposures could occur both for the fetal loss and congenital anomaly cohorts. This was to investigate the presence or absence of a drug pre-conception effect.

#### Classifying Category C Drugs Into Harmful and Non-Harmful Pregnancy Categories

We only investigated drugs with at least 50 pregnancies across all five-exposure periods (e.g., first trimester, second trimester) to minimize statistical anomalies due to low data. We excluded all drugs classified as FDA pregnancy category N (i.e., Not Classified) or drugs that were ‘Not Listed’. For visualization purposes, we performed Multi-Dimensional Scaling (MDS) component analysis to assess the relationship between the proportion of fetal loss (or proportion with congenital anomaly depending on the model) per trimester of exposure to illustrate the relationship between adverse fetal outcomes and FDA pregnancy category. We also visualized only drugs known to be prescribed with legal termination to determine where in each of the visualizations those drugs appeared.

#### Logistic Regression

We first performed a logistic regression model to predict a binary pregnancy category either ‘Detrimental to Fetus – D or X’ or ‘Not Harmful to Fetus – A or B’. We built three models – one model for fetal loss, a second for congenital anomalies, and a third for minor congenital anomalies only. This allowed us to determine Odds Ratios (OR) and significance in a full model. The full model includes 29 features: one for each of 14 ATC classifications, 5 features indicating the number exposed during each trimester category (3 trimesters plus two 3-month periods for the pre-conception period), 5 features indicated the proportion of exposed with an anomaly per trimester category, 1 binary indicator variable for whether Mendelian genes were inhibited (from OMIM), 1 binary indicator variable whether Mendelian genes were affected (from OMIM), one binary indicator variable for whether vitamin genes are affected (from DisGeNET), one binary indicator variable for whether or not the drug could be used as a prenatal supplement (e.g., vitamin, mineral, glucose), and one binary indicator variable for whether or not the drug was a treatment for nicotine abuse (since exposure to smoking during the prenatal period is a known risk factor for fetal loss and anomalies). For the fetal loss model, we only had 25 features because the majority of fetal losses occurred during the first trimester and therefore we did not have variables for second and third trimester (either proportion of anomalies or exposed).

#### Random Forest Classifier

We constructed a random forest model to classify both fetal loss and congenital anomalies (separately) with 2000 trees using all possible features. Out-Of-Bag (OOB) error rates were estimated to assess the quality of each model. Features were ranked using the Mean Decrease in Accuracy (MDA) with more informative features having higher MDAs. This allowed us to assign probabilities for each drug as being harmful (similar to a category D or X drug) or safe (similar to a category A or B drug). We compared a drug’s probability of being harmful from each model for drugs with known FDA status and those with no recommendation (i.e., FDA category C drugs). Code was implemented using R version 3.3.0.

## Electronic supplementary material

Supplemental Information

Supplemental Dataset 1

Supplemental Dataset 2

Supplemental Dataset 3

Supplemental Dataset 4
