# Supplementary material for: Development of A Machine Learning Algorithm to Classify Drugs Of Unknown Fetal Effect
Source: Sci Rep. 2017 Oct 9;7:12839. doi: 10.1038/s41598-017-12943-x (PMC5634437; doi:10.1038/s41598-017-12943-x)
Supplement: Supplementary file 1 — Supplemental Information [file 41598_2017_12943_MOESM1_ESM.pdf]

Supplemental Material for:

Boland MR, Polubriaginof F, Tatonetti NP.

Development of a Machine Learning Algorithm to Classify Drugs Of Unknown Fetal Effect

Table of Contents

Figures……………………………………………………………………………………………….2

Figure S1. Odds Ratios from Logistic Regression Models: Fetal Loss, Congenital Anomaly and Minor Congenital Anomaly……………………………………………………………………………………2

Figure S2. Component vs. Proportion with Congenital Anomaly Following First Trimester Drug Exposure…...3

Figure S3. Component vs. Proportion with Congenital Anomaly Following Second Trimester Drug Exposure...4

Figure S4. Component vs. Proportion with Congenital Anomaly Following Third Trimester Drug Exposure…..5

Figure S5. Component vs. Proportion with Minor Congenital Anomalies Only Following First Trimester Drug Exposure…………………………………………………………………………………………......6

Figure S6. Component vs. Proportion with Minor Congenital Anomalies Only Following Second Trimester Drug Exposure……………………………………………………………………………………………7

Figure S7. Component vs. Proportion with Minor Congenital Anomalies Only Following Third Trimester Drug Exposure…………………………………………………………………………………………......8

Figure S8. Mean Decrease in Accuracy (MDA) Plots for: Fetal Loss, Congenital Anomaly and Minor Congenital Anomaly………………………..9

Tables………………………………………………………………………………………………10

Table S1. Common Fetal Loss Diagnoses (having at least 100 Pregnancies) Included In Our Study…..………10

Table S2. FDA Pregnancy Categories and Descriptions………………………..…………………………11

Table S3. ATC Classifications and Descriptions……………………………………..………………….12

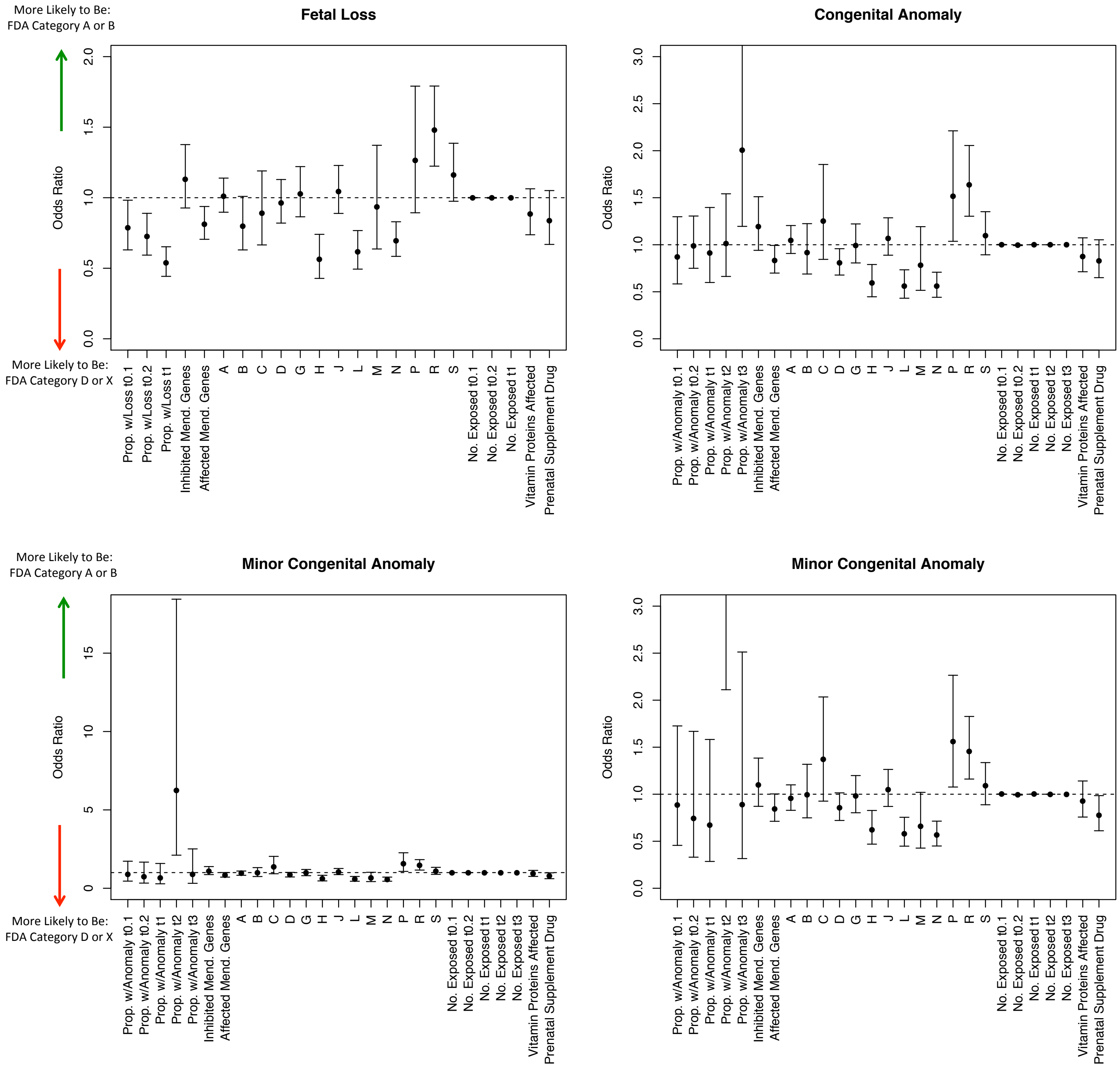

Figure S1. Odds Ratios from Logistic Regression Models: Fetal Loss, Congenital Anomaly and Minor Congenital Anomaly. ORs less than 1 indicate that the model predicts that feature to indicate that a drug is more likely to be a FDA Category D or X Drug. ORs greater than 1 indicate that the model predicts that feature to indicate that a drug is more likely to be a FDA Category A or B drug. In the fetal loss model, a drug having an ATC category of R (Respiratory System drug) increases the probability that the drug is an FDA Category A or B drug (i.e., a ‘good’ drug) while an ATC category of H (Systemic Hormonal Preparations excluding sex hormones and insulins) increases the probability that the drug is FDA Category D or X (i.e., a ‘harmful’ drug). The diagram on the lower right-hand quadrant depicts the ORs for the minor congenital anomaly model (same as lower lefthand-quadrant) except that the range is tightened (cannot see the one outlier Prop. w/anomaly t2) to illustrate the variability among the other model predictors minimizing the outlier effect on the visualization.

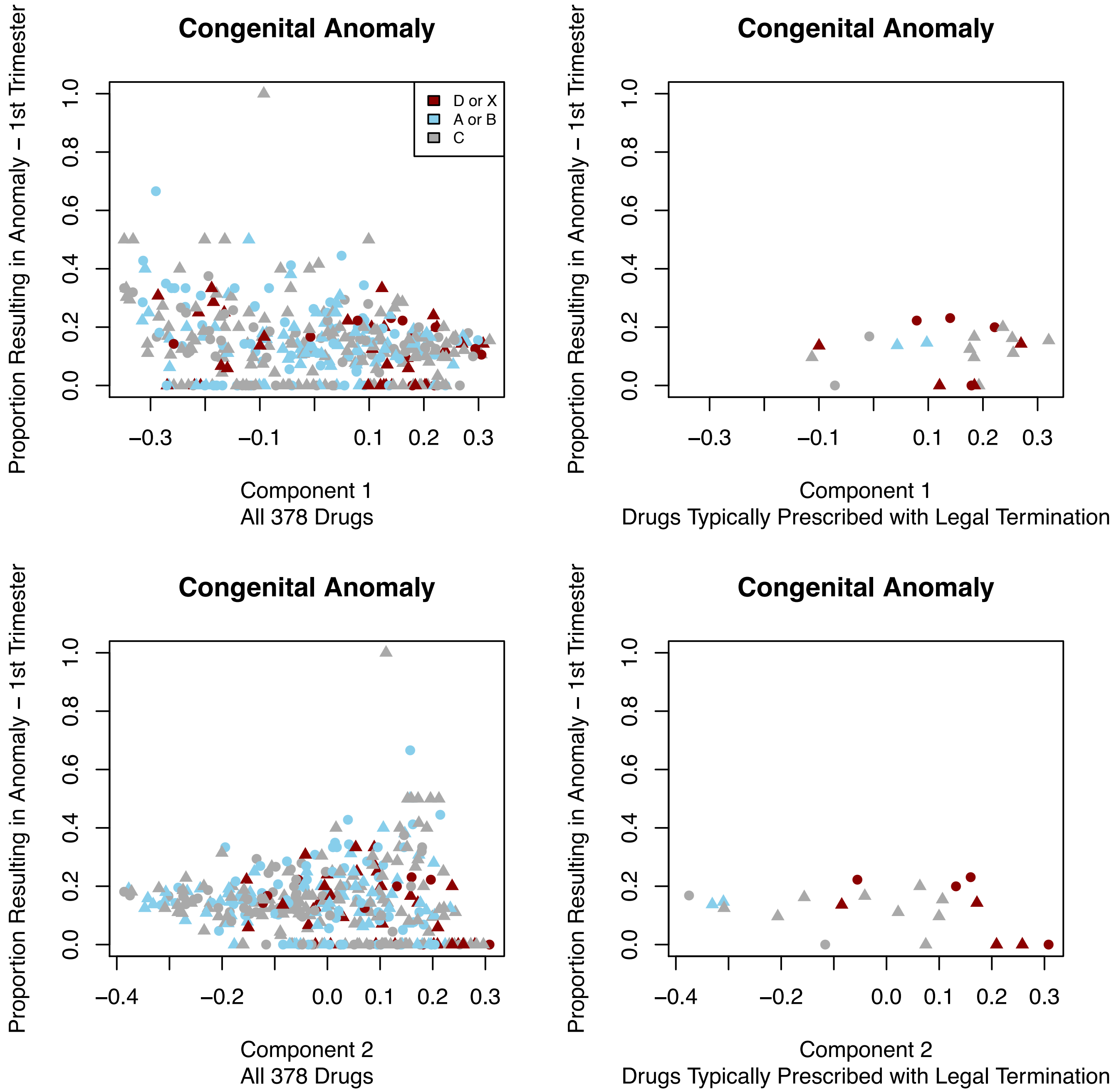

Figure S2. Component vs. Proportion with Congenital Anomaly Following First Trimester Drug Exposure. The relationship between the first component and the proportion of individuals experiencing a congenital anomaly following prenatal exposure to the drug during the first trimester. This effect is not entirely due to drugs prescribed for legal termination, which are shown separately in the right most subplots.

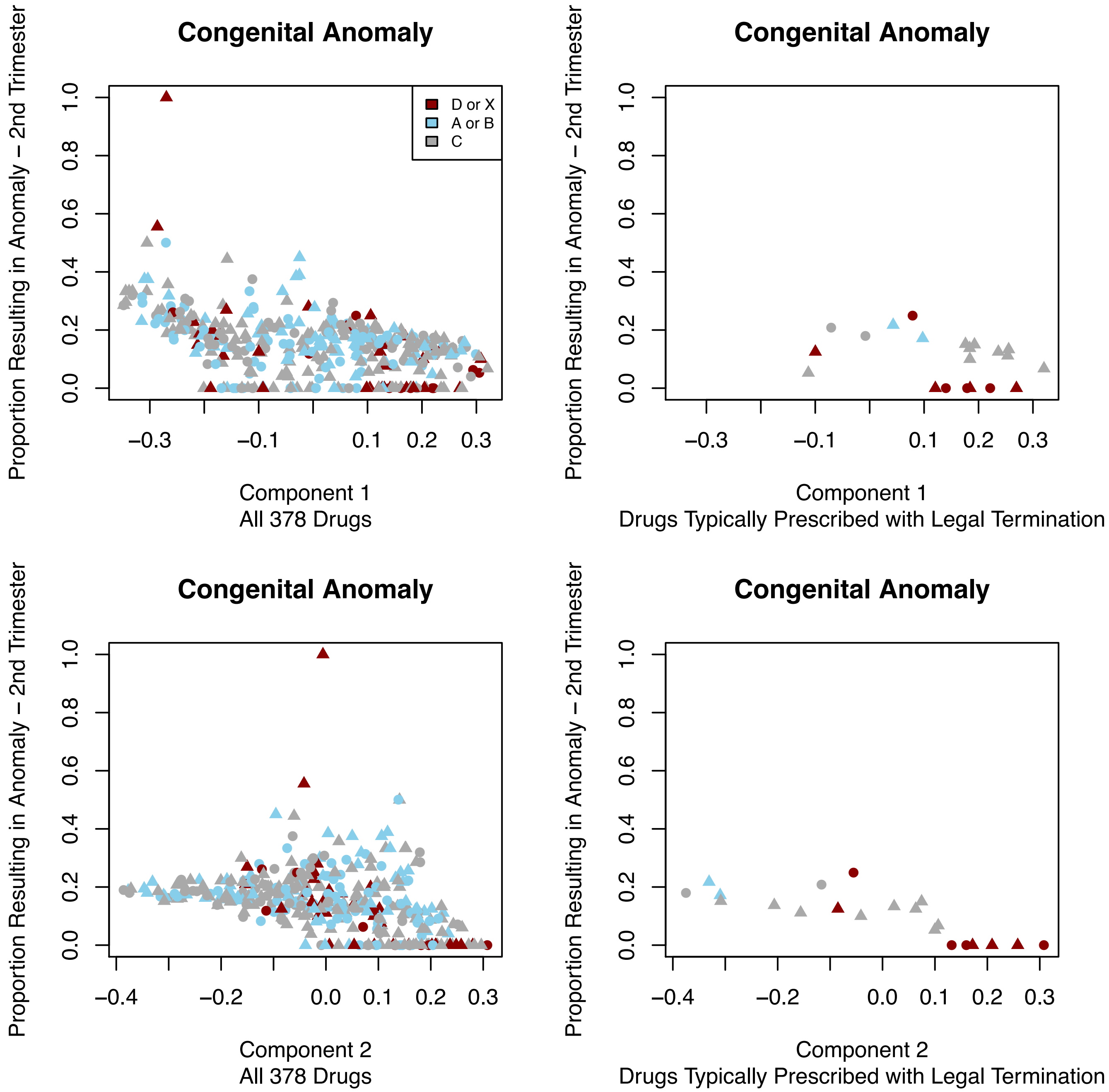

Figure S3. Component vs. Proportion with Congenital Anomaly Following Second Trimester Drug Exposure. The relationship between the first component and the proportion of individuals experiencing a congenital anomaly following prenatal exposure to the drug during the second trimester. This effect is not entirely due to drugs prescribed for legal termination, which are shown separately in the right most subplots.

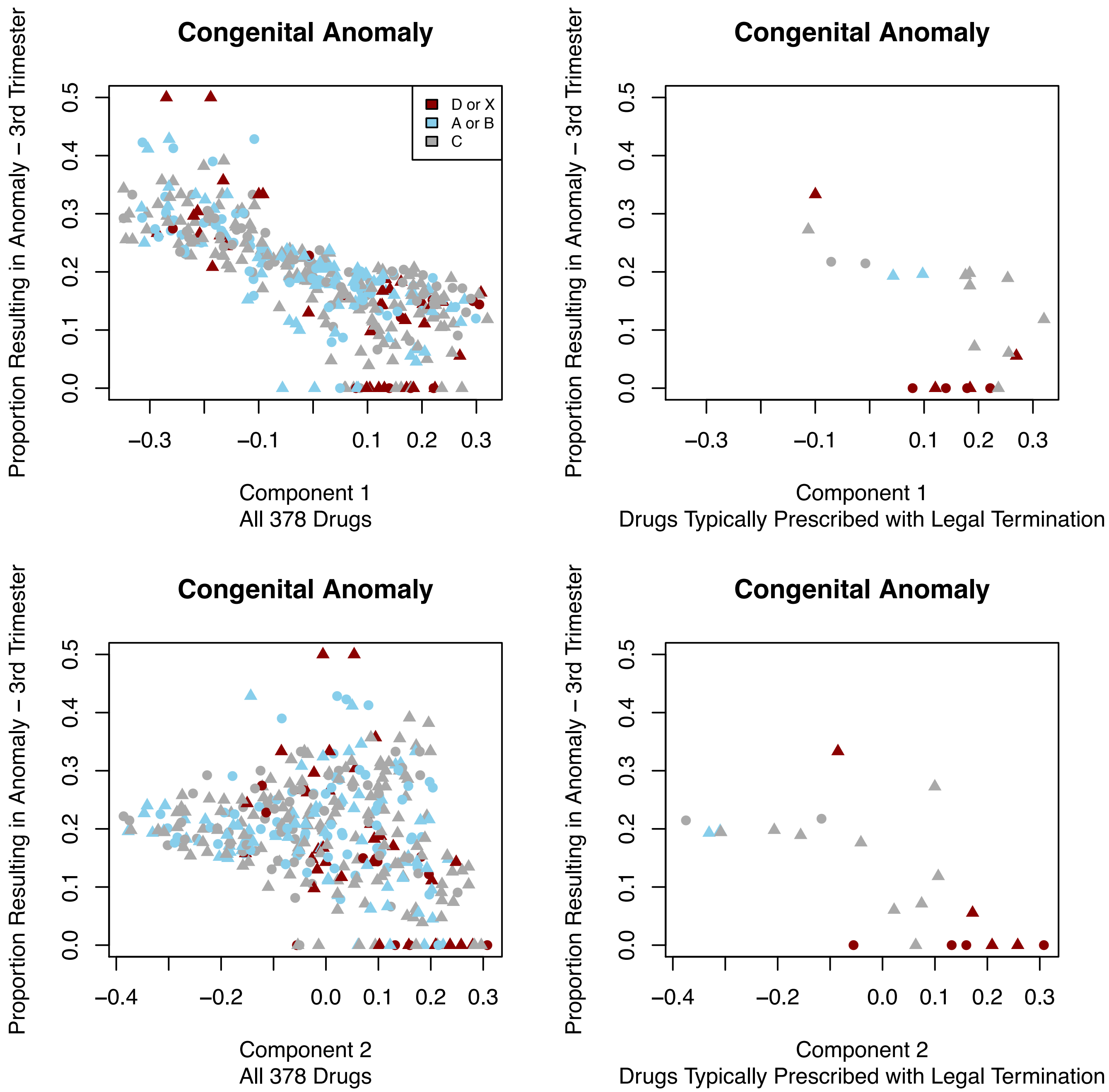

Figure S4. Component vs. Proportion with Congenital Anomaly Following Third Trimester Drug Exposure. The relationship between the first component and the proportion of individuals experiencing a congenital anomaly following prenatal exposure to the drug during the third trimester. This effect is not entirely due to drugs prescribed for legal termination, which are shown separately in the right most subplots.

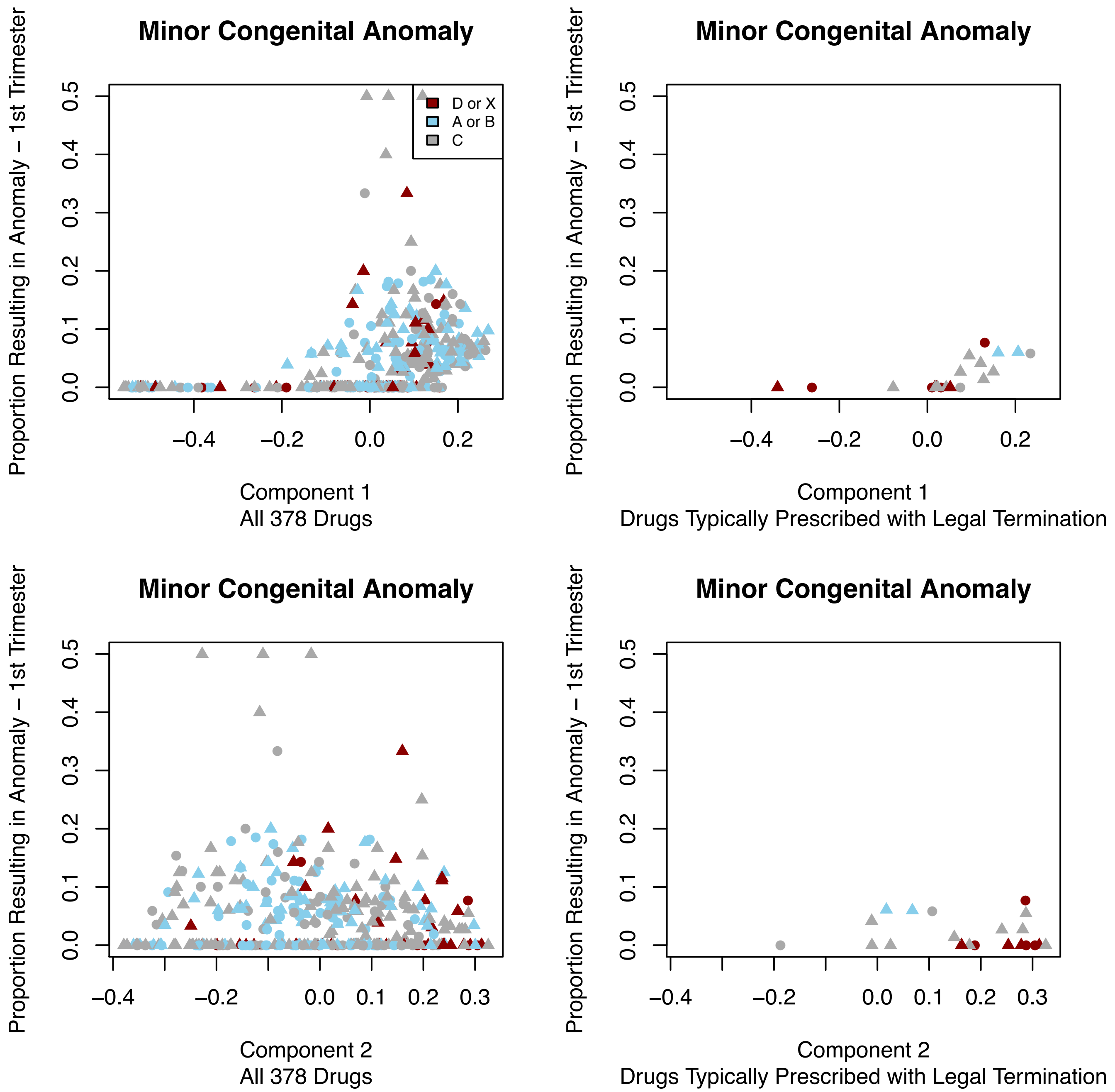

Figure S5. Component vs. Proportion with Minor Congenital Anomalies Only Following First Trimester Drug Exposure. The relationship between the first component and the proportion of individuals experiencing a minor congenital anomaly following prenatal exposure to the drug during the first trimester. This effect is not entirely due to drugs prescribed for legal termination, which are shown separately in the right most subplots.

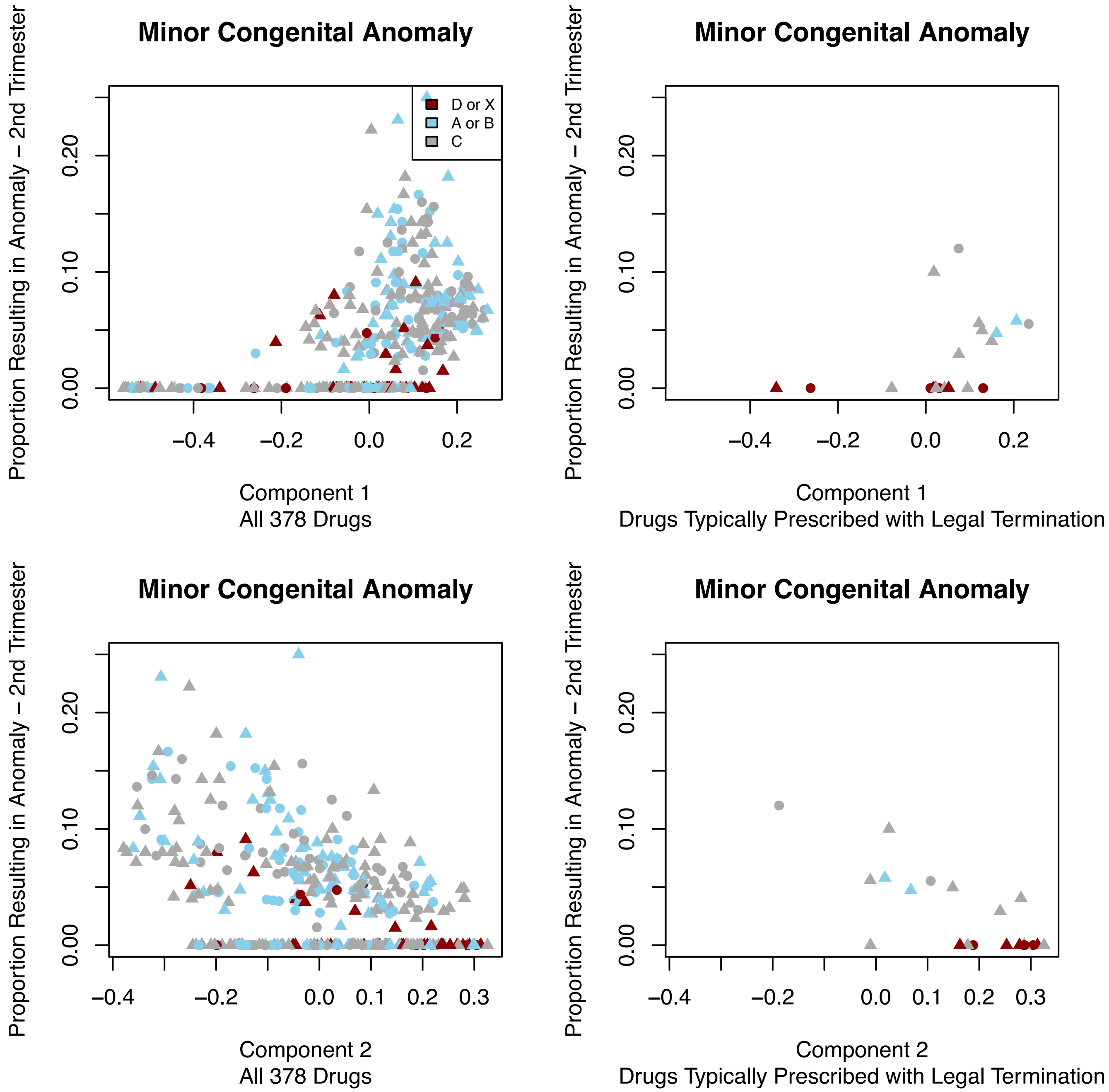

Figure S6. Component vs. Proportion with Minor Congenital Anomalies Only Following Second Trimester Drug Exposure. The relationship between the first component and the proportion of individuals experiencing a minor congenital anomaly following prenatal exposure to the drug during the second trimester. This effect is not entirely due to drugs prescribed for legal termination, which are shown separately in the right most subplots.

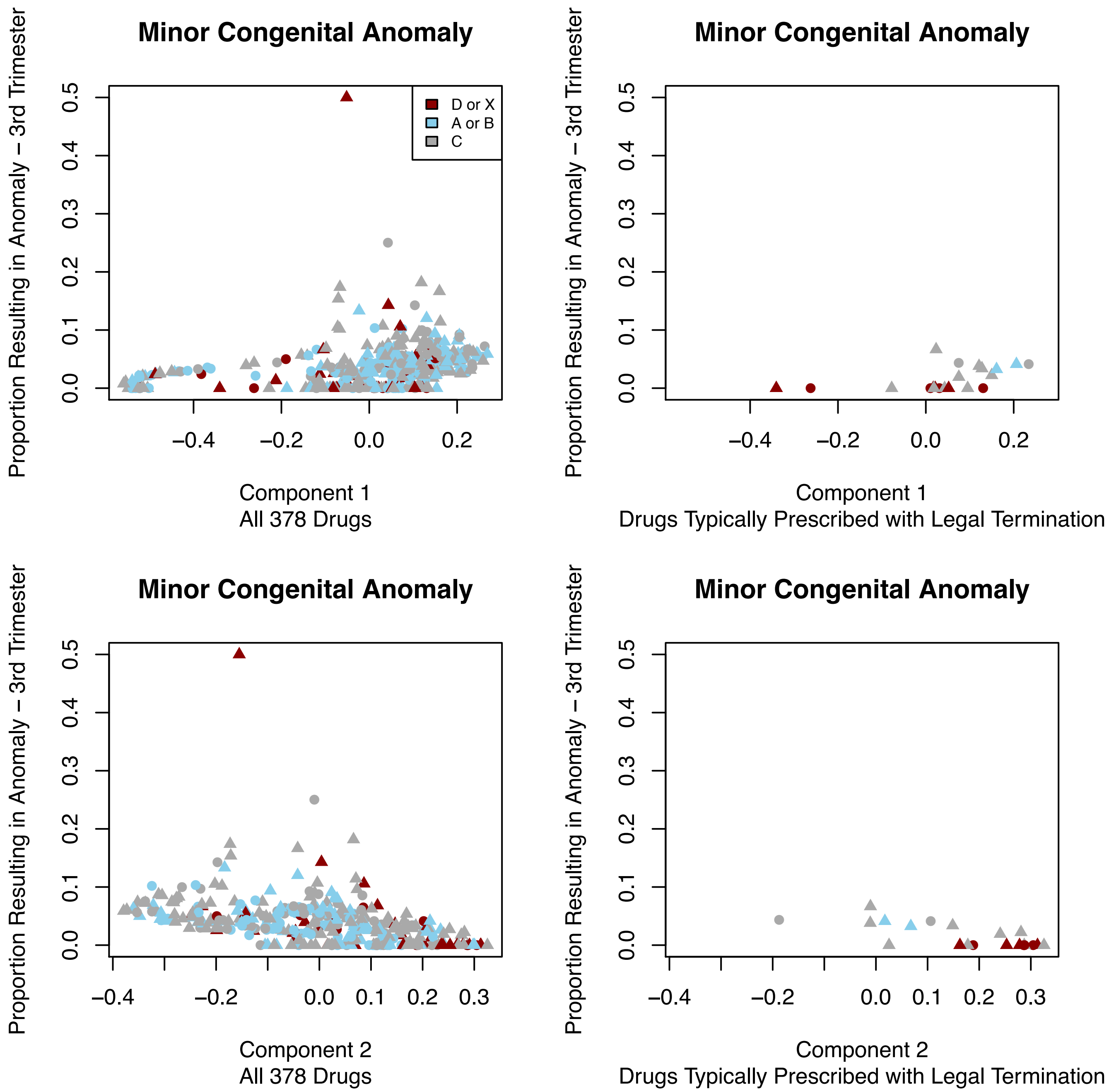

Figure S7. Component vs. Proportion with Minor Congenital Anomalies Only Following Third Trimester Drug Exposure. The relationship between the first component and the proportion of individuals experiencing a minor congenital anomaly following prenatal exposure to the drug during the third trimester. This effect is not entirely due to drugs prescribed for legal termination, which are shown separately in the right most subplots

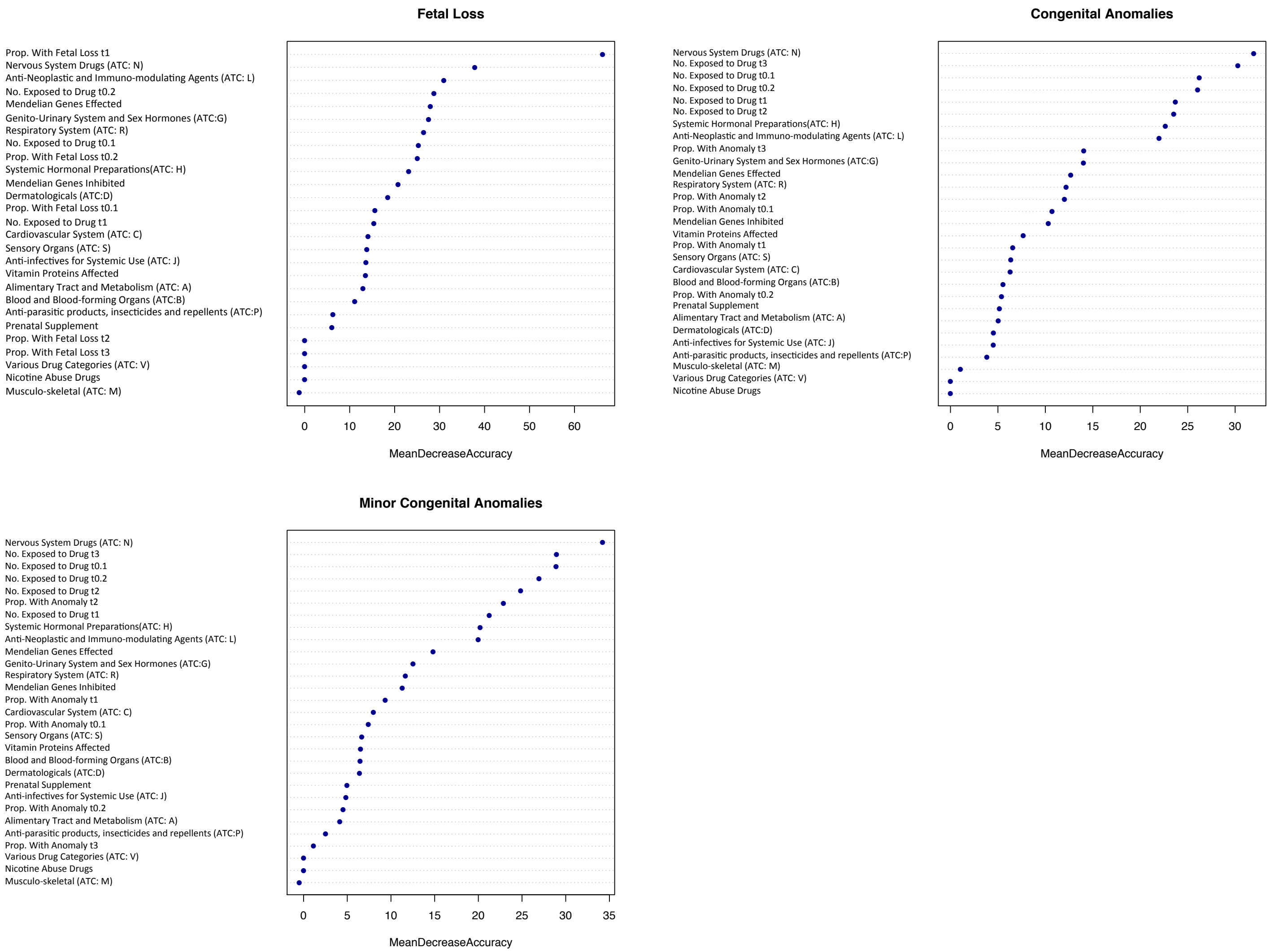

Figure S8. Mean Decrease in Accuracy (MDA) Plots for: Fetal Loss, Congenital Anomaly and Minor Congenital Anomaly. The higher the MDA score, the more informative the feature is to the model's performance. This means that features towards the top of each subplot are the most indicative of whether a drug is known to be harmful (D or X) versus not-known-to-be-harmful (A or B). The number of individuals exposed to a drug at each trimester was highly informative of the drug's pregnancy class (i.e., harmful (D or X) or not-known-to-be-harmful (A or B)). This is intuitive as physicians often modify their behavior when they identify a woman as being pregnant and are less likely to give them a known harmful drug (i.e., D or X). ATC drug class N is also very predictive in all 3 models, but was more predictive for the congenital anomalies models then the fetal loss model.

Table S1. Common Fetal Loss Diagnoses (having at least 100 Pregnancies) Included In Our Study

ICD-9 Code	ICD-9 Name	Number of Distinct Pregnancies
635.92	Legally induced abortion, without mention of complication, complete	4896
635.90	Legally induced abortion, without mention of complication, unspecified	2357
632	Missed abortion	1922
635.91	Legally induced abortion, without mention of complication, incomplete	1418
634.90	Spontaneous abortion, without mention of complication, unspecified	1256
633.10	Tubal pregnancy without intrauterine pregnancy	879
633.90	Unspecified ectopic pregnancy without intrauterine pregnancy	867
634.92	Spontaneous abortion, without mention of complication, complete	833
634.91	Spontaneous abortion, without mention of complication, incomplete	745
637.91	Unspecified abortion, without mention of complication, incomplete	571
637.92	Unspecified abortion, without mention of complication, complete	419
633	Ectopic pregnancy	342
637.90	Unspecified abortion, without mention of complication, unspecified	269
630	Hydatidiform mole	247
639.1	Delayed or excessive hemorrhage following abortion or ectopic and molar pregnancies	160
633.01	Abdominal pregnancy with intrauterine pregnancy	145
639	Complications following abortion and ectopic and molar pregnancies	142

Table S2. FDA Pregnancy Categories and Descriptions

FDA Category	Description	Studies In Humans	Studies In Animals	No. of Drugs in Fetal Loss Dataset (N=499)*	No. of Drugs in Congenital Anomaly Dataset (N=378)*
A	No Risk in Controlled Human Studies	No Risk To Fetus	No Risk To Fetus	20	15
B	No Risk in Other Studies	No Adequate Studies<br>OR<br>No Risk To Fetus	No Risk To Fetus<br><br>**Risk To Fetus**	147	125
C	Risk Not Ruled Out	No Adequate Studies	**Risk To Fetus**	264	192
D	Positive Evidence of Risk	**Risk To Fetus**	**Risk To Fetus**	37	26
X	Contraindicated in Pregnancy	**Risk To Fetus** – Including Anomalies	**Risk To Fetus**	31	20
N	Not Yet Classified Into A Pregnancy Category	-	-	-	
Not Listed		-	-	-	

***Distinct Drug-Dosage Combos – A drug only has 1 FDA Pregnancy Classification**

Table S3. ATC Classifications and Descriptions

ATC Category	Description	No. of Drugs in Fetal Loss Dataset*	No. of Drugs in Congenital Anomaly Dataset*
A	Alimentary Tract and Metabolism	131	113
B	Blood and Blood-Forming Organs	44	38
C	Cardiovascular System	53	41
D	Dermatologicals	81	65
G	Genito-Urinary System and Sex Hormones	81	61
H	Systemic Hormonal Preparations, excluding sex hormones and insulins	21	18
J	Anti-infectives for Systemic Use	69	58
L	Anti-neoplastic and Immuno-modulating agents	15	10
M	Musculo-skeletal System	17	11
N	Nervous system	72	44
P	Anti-parasitic products, insecticides and repellents	6	6
R	Respiratory System	76	53
S	Sensory Organs	60	49
V	Various	12	12

***Distinct Drug-Dosage Combos – A drug can have multiple ATC classifications**
